# Genome-Wide Prediction and Validation of Peptides That Bind Human Prosurvival Bcl-2 Proteins

**DOI:** 10.1371/journal.pcbi.1003693

**Published:** 2014-06-26

**Authors:** Joe DeBartolo, Mikko Taipale, Amy E. Keating

**Affiliations:** MIT Department of Biology, Cambridge, Massachusetts, United States of America; Fox Chase Cancer Center, United States of America

## Abstract

Programmed cell death is regulated by interactions between pro-apoptotic and prosurvival members of the Bcl-2 family. Pro-apoptotic family members contain a weakly conserved BH3 motif that can adopt an alpha-helical structure and bind to a groove on prosurvival partners Bcl-x_L_, Bcl-w, Bcl-2, Mcl-1 and Bfl-1. Peptides corresponding to roughly 13 reported BH3 motifs have been verified to bind in this manner. Due to their short lengths and low sequence conservation, BH3 motifs are not detected using standard sequence-based bioinformatics approaches. Thus, it is possible that many additional proteins harbor BH3-like sequences that can mediate interactions with the Bcl-2 family. In this work, we used structure-based and data-based Bcl-2 interaction models to find new BH3-like peptides in the human proteome. We used peptide SPOT arrays to test candidate peptides for interaction with one or more of the prosurvival proteins Bcl-x_L_, Bcl-w, Bcl-2, Mcl-1 and Bfl-1. For the 36 most promising array candidates, we quantified binding to all five human receptors using direct and competition binding assays in solution. All 36 peptides showed evidence of interaction with at least one prosurvival protein, and 22 peptides bound at least one prosurvival protein with a dissociation constant between 1 and 500 nM; many peptides had specificity profiles not previously observed. We also screened the full-length parent proteins of a subset of array-tested peptides for binding to Bcl-x_L_ and Mcl-1. Finally, we used the peptide binding data, in conjunction with previously reported interactions, to assess the affinity and specificity prediction performance of different models.

## Author Summary

Bcl-2 family proteins regulate key cell death vs. survival decisions and are implicated in the development of many cancers. To understand the roles of Bcl-2 family proteins in both normal and diseased cells, it is important to map the interaction network of the family. Low sequence conservation in known Bcl-2 interaction motifs precludes easy identification of possible binding partners, but we developed computational models based on structure and experimental mutation data that show good predictive performance. We used our models to search the human proteome for new Bcl-2 interaction partners. We predicted and experimentally validated more than twice as many tight-binding peptides as were previously known.

## Introduction

Bcl-2 family proteins regulate programmed cell death and play key roles in eukaryotic development and in the onset and progression of many human diseases, including cancer [1], [2]. Intensive efforts to develop therapeutic agents that target Bcl-2 family members underscore their importance [3]. The family consists of three groups of proteins: prosurvival Bcl-2 proteins (Bcl-x_L_, Bcl-2, Bcl-w, Mcl-1 and Bfl-1); pro-apoptotic death effectors (Bak, Bax, Bok); and ∼10 Bcl-2 homology 3 (BH3) motif-only members (Bim, Bad, Puma, etc.). The five prosurvival proteins share a common fold [4]–[9], and all family members contain a BH3 motif that can adopt a helical structure of ∼18–26 residues. Biophysical and structural studies have established that peptides corresponding to BH3 motifs dock in a hydrophobic groove on the surface of prosurvival proteins, which we refer to as “receptors” [4]–[9]. All receptors, however, do not interact with all BH3 peptides. For example, Bad BH3 interacts strongly with Bcl-x_L_, Bcl-w and Bcl-2, but not Mcl-1 or Bfl-1, and Noxa BH3 only interacts strongly with Mcl-1 [10]–[14]. Competitive binding of BH3 helices from sensitizers vs. activators vs. death effectors to prosurvival proteins appears critical for regulating cell death via apoptosis [13].

BH3-only proteins have been discovered over the past two decades using varied methods, including yeast two-hybid assays and phage/cDNA-based screening assays [15]. Additional BH3-only proteins may remain undiscovered, especially proteins that are co-expressed and/or co-localize with prosurvival Bcl-2 receptors only in certain cell types, intracellular compartments or developmental stages. Identifying a complete set of interaction partners is important for building a full understanding of the functional roles of Bcl-2 receptors.

BH3-only proteins vary in sequence, structure and function, and many share in common only the BH3 motif. Discovery of new BH3-only proteins by sequence profile analysis is confounded by the fact that known BH3 motifs are short (∼23 residues) and can have as few as 3 residues in common. Aoucheria and coworkers have discussed how sequence analysis has led to both misidentification and overestimation of potential BH3 motifs [15]. Pfam annotates the BH3 alignments of orthologs of individual BH3-only proteins, but contains no universal BH3 class [16]. Prosite defines a universal BH3 motif class and provides an incomplete list of ∼10 unique BH3 motifs from known Bcl-2 proteins and their isoforms [17]. When the three most conserved BH3 residues (L-X-X-X-σ-D, where σ is A, G, S) are specified, there are still more than 10,000 potential human peptide matches in the genome. Moreover, recent work has shown that BH3 peptide binders can tolerate significantly more sequence diversity than suggested by the sequences of the small number of known natural binders [18]–[20]. In particular, Dutta et al. used peptide SPOT arrays and yeast-surface display library screening to identify large numbers of mutations in the BH3 region of Bim that maintain binding to pro-survival receptors Bcl-x_L_ and/or Mcl-1 [20]. Similar results were found for Bfl-1, Bcl-2 and Bcl-w [18], [19], [21].

Recently, experimentally verified binders have been used to construct simple models to score the binding of BH3-like peptides. For example, published PSSM_SPOT_ models are based on the SPOT-array signal intensities for 180 single-residue mutants of Bim BH3 binding to different receptors [19], [20]. Structure-based methods including the Rosetta FlexPepBind protocol and the statistical potential STATIUM can also be used to predict BH3-peptide binding, with accuracy similar to that of the PSSM_SPOT_ models [19], [21]. Here, we report the results of using PSSM_SPOT_ and STATIUM models to identify new putative BH3 motifs in human proteins. We searched for peptides predicted to interact with any of the five Bcl-2 prosurvival receptors, with an emphasis on identifying new binders of Mcl-1, Bcl-x_L_ and Bfl-1. We tested many predicted interactions using SPOT arrays and then measured the Bcl-2 family binding specificity of the best hits using solution-phase binding experiments. Finally, we assembled a large and diverse dataset of BH3-peptide binding data, including new interactions reported here, to benchmark the affinity and specificity prediction performance of different models.

## Results

### Improved structure-based prediction of Bcl-2 interactions using STATIUM_SC_

**Figure 1:**
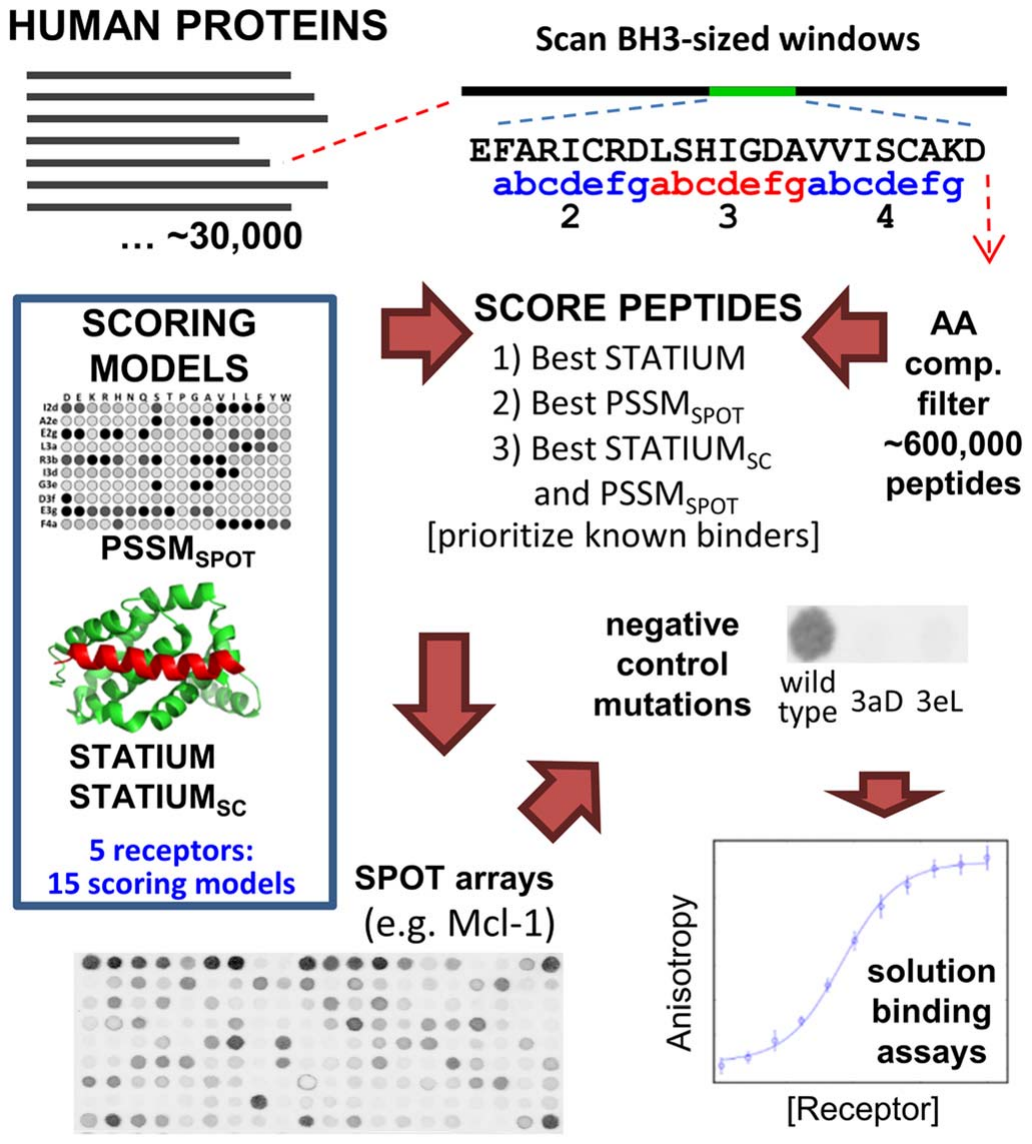
Prediction and validation of BH3-like peptides. Proteins from the Human Protein Reference Database were scanned in 23-residue windows, sequentially aligning each window with the [abcdefg]_n_ heptad definition of a BH3 motif, as defined in the figure. Sequences were then filtered for amino-acid composition to give ∼600,000 candidate peptide sequences to be evaluated [46]. Each sequence was scored with STATIUM, STATIUM_SC_, and PSSM_SPOT_ models for binding to each of the 5 prosurvival proteins Bcl-x_L_, Bcl-w, Bcl-2, Mcl-1 and Bfl-1 (15 scores in all, for each sequence), and candidate BH3-like sequences with good scores were selected for testing on SPOT arrays, as described in the Methods. A subset of peptides with successful negative controls on the SPOT arrays was tested for binding in solution. PSSM_SPOT_ cartoon is for demonstration: See Methods for the references to data used to derive the model.

STATIUM is a structure-specific scoring function that can be used to evaluate the fit of a sequence on a structural template. The original version of STATIUM used only the locations of the Cα and Cβ atoms of the template to derive the potential [19]. We have now implemented a new version of STATIUM for modeling protein complexes that uses an all-heavy-atom representation of the receptor residues in a receptor-peptide complex, but maintains the Cα/Cβ-only description of the peptide residues. This version is appropriate for applications such as genome scanning, in which the sequence of the receptor is fixed while that of the peptide is varied. We were motivated to include the additional atoms when we observed that receptor side-chain configurations are similar in structures of the same receptor bound to different peptides (see SI Text and Table S1). The full-side chain version of STATIUM, which we refer to as STATIUM_SC_, is described in detail in the Methods. STATIUM_SC_ scores known BH3 motifs (Bim, Puma, etc.) better than STATIUM. The comparison we use is the average Z-score of known binders based on the distribution of scores for ∼600,000 BH3-sized sequence frames in the human proteome (see Methods). The average Z-score for known binders is higher for STATIUM_SC_ than for the original STATIUM for all five receptors (Bcl-x_L_: 2.1 vs. 0.98 for 7 BH3 sequences, Mcl-1: 3.1 vs. 1.1 for 7 BH3 sequences, Bcl-w: 2.4 vs. 1.3 for 7 BH3 sequences, Bcl-2: 2.6 vs. 1.2 for 6 BH3 sequences, Bfl-1: 2.5 vs. 2.1 for 3 BH3 sequences). In addition, STATIUM_SC_ scored the strictly conserved aspartic acid at BH3 position 3f as the top amino acid for all receptor models, whereas original STATIUM had an equally strong preference for other hydrophilic residues at this site. BH3 position numbering in this paper follows a heptad convention [abcdefg]_n_ that describes the hydrophobic/polar patterning, with ‘a’ and ‘d’ positions typically hydrophobic, and with the core of the motif corresponding to 12 residues labeled as 2d, 2e, 2f, …, 4a, see Figure 1. Further comparisons that demonstrate the enhanced performance of STATIUM_SC_ and STATIUM follow below.

### PSSM_SPOT_

In addition to STATIUM and STATIUM_SC_, we used the previously reported, experimental data-based PSSM_SPOT_ model to score interactions between BH3-like peptides and Bcl-2 receptors [19], [20]. PSSM_SPOT_ models for each anti-apoptotic protein were derived from experiments in which point mutants of Bim BH3 peptides were tested for binding to each receptor using SPOT arrays. The PSSM_SPOT_ score for a residue is computed as the logarithm of the SPOT array signal (as a fraction of the wild-type Bim BH3 signal) for that amino acid substituted at the corresponding position in Bim (See Methods). Residue scores are summed over ten positions for which experimental data are available to obtain peptide scores. Thus, PSSM_SPOT_ can only evaluate binding contributions at ten positions in the core of a BH3 peptide (positions 2d, 2e, 2g, 3a, 3b, 3d, 3e, 3f, 3g, 4a), whereas STATIUM can model any peptide position involved in an interaction with the receptor in the structure of a complex. STATIUM, STATIUM_SC_ and PSSM_SPOT_ are all receptor-specific models, meaning that a different model is derived and applied to predict binding to each prosurvival receptor protein.

### SPOT array positive and negative controls

We used peptide SPOT arrays as part of our pipeline for identifying candidate new BH3 peptides. In an initial assessment of the performance of the SPOT array assay for detecting Bcl-2 protein-peptide interactions, we tested 8 positive control peptides (Corresponding to BH3 motifs from Bim, Puma, Noxa, Bad, Bik, Bid, Bmf and Hrk) [10]. We used Bim BH3 as a strong-binding reference, because Bim BH3 26-mers bind to all receptors with low-nanomolar affinity in solution [20]. SPOT array signals for positive controls ranged from 76% to 209% of the Bim BH3 peptide signal. Negative control mutations at two of the most conserved BH3 positions were used to provide information about peptide binding mode. Position 3a is strongly conserved as Leu and is buried at the BH3-receptor interface [22]–, so we mutated this position to Asp in candidate peptides as a negative control. Position 3e is restricted to small residues in known BH3 motifs and packs tightly against the receptor [22]–[24], so we mutated this position to Leu in candidate peptides as a separate negative control. The SPOT array signal was reduced by 62–99% compared to the wild-type peptide for the 3aD mutation among 8 positive control peptides, and by 57–95% for the 3eL mutation. Known weak interactions/non-binders gave very low signals on the arrays, or occasionally gave higher signals that were not reduced by negative control mutations. E.g. a peptide from BNIP3L previously reported to not bind any receptor tightly (five K_D_ values over 2.5 µM for all five receptors [10]) had a signal of less than 3% of Bim BH3 for binding to all receptors except Bfl-1 (Bfl-1 binding gave 8% of the Bim BH3 signal). Bad BH3 interactions with Mcl-1 and Bfl-1 (both K_D_>2.5 µM [10]) gave 48–52% of the Bim BH3 signal, but these signals were reduced by less than 25% for at least one of the negative controls. Additional information about the performance of the SPOT arrays for newly identified peptides is reported below.

### Prediction and screening of new BH3-like peptide binders

We used STATIUM, STATIUM_SC_ and PSSM_SPOT_ to search for new BH3-like peptides in the human proteome by scoring sliding BH3-length (23-residue) windows corresponding to positions 1g to 5a (Figure 1). We reduced the pool of candidates to those that matched the compositional profile of known BH3 motifs (see Methods), resulting in 591,829 candidate peptide sequences. This set of sequences was scored with each of our 15 models (3 models each for 5 prosurvival receptors). We used the scores, and protein functional annotations, to prioritize peptides for testing on SPOT arrays as described below and, in more detail, in the Methods.

SPOT arrays of computationally predicted BH3 peptides were designed and tested in 5 sequential experiments, with solution binding experiments used to test candidate binders between rounds (for details of what was tested in each array experiment, see Methods and Table S2). Our first three arrays tested top-scoring interactions according to STATIUM and PSSM_SPOT_, prior to the development of STATIUM_SC_. For these experiments, we prioritized top-scoring peptides, particularly those within proteins reported to interact with Bcl-2 receptors or their interaction partners (see Methods). A total of 560 peptides were tested on the first three arrays for interaction with one or more of the five prosurvival proteins. All predicted BH3 peptides were tested for binding to all five pro-survival proteins on array I. On arrays II and III, new candidate peptides were tested for binding to Bcl-x_L_ and/or Mcl-1. Out of a total of 1150 interactions tested, 504 interactions gave a signal that was at least 5% of the wild-type Bim BH3 signal (373>10%, 220>25%, Table S2). 244 interactions were tested with the two negative control mutations described above. A reduction in signal of at least 30% for two negative control mutations was observed for 54 of those interactions. We found these negative control cutoffs useful for identifying real interactions, as discussed below, and in analysis we designated interactions that passed these cutoffs as “candidate array interactions.”

STATIUM_SC_ was developed after the first three array experiments were completed. Using the data from those experiments, we observed that a combination of STATIUM_SC_ and PSSM_SPOT_ scores was better at identifying binders from arrays I–III than was either scoring method alone. Specifically, we observed that interactions tested on the array that had both PSSM_SPOT_ and STATIUM_SC_ Z-scores better than 2.0 were more enriched in candidate array interactions than interactions with only PSSM_SPOT_ or STATIUM_SC_ Z-scores greater than 2.0 (39% vs. 24% or 32%, Table S2). Thus, we synthesized a final array of peptides (array IV) that had top STATIUM_SC_ scores and also passed a PSSM_SPOT_ threshold (see Methods, and Supplementary Table S2 and Data S1 for array data). 38% of all peptides tested on array IV participated in a candidate array interaction with at least one prosurvival protein (41 peptides), compared to 28% for all peptides tested with negative controls on the previous arrays (43 peptides). The fourth array would also have included 74% of all peptides that participated in previously identified candidate array interactions, had these not already been tested. 17 peptides that participated in candidate array interactions on arrays I or III, and 19 peptides from array IV, were tested for binding to 5 prosurvival proteins in solution.

**Figure 2:**
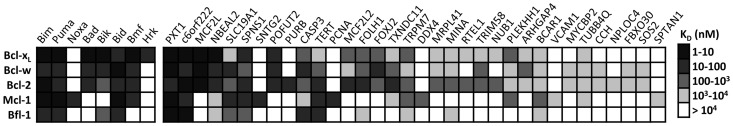
Bcl-2 receptor binding profiles of 36 BH3-like peptides from human proteins. Binding profiles for known BH3 peptides interacting with Bcl-x_L_, Bcl-w, Bcl-2, Mcl-1 and Bfl-1, measured by Certo et al., are in the left panel [10]. 34 peptides identified in this study with K_D_<10^4^ nM for binding to at least one of five prosurvival proteins are in the right panel; these are ordered from left to right according to binding affinity, as indicated in the greyscale key. See Table S3 for the K_D_ values used for binning and 95% confidence intervals.

Figure 2 reports the affinities of interactions that we measured among 34 peptides and 5 prosurvival proteins; two of the 36 peptides tested showed very weak binding in solution (Table S3, Figure S1). 52 interactions had K_D_<500 nM and 102 had K_D_<5 µM. Most peptides derived from known BH3 motifs with lengths 20–26 residues are reported to bind to receptors with affinities ranging from ∼1–350 nM [4], [10], [12], and we refer to new interactions with K_D_<500 nM as “tight interactions” below. All solution-validated peptides competed with Bim BH3 for binding to the prosurvival proteins, consistent with the structural model underlying our predictions (Figure S2). 94% of the peptides that formed tight interactions in solution met the STATIUM_SC_ and PSSM_SPOT_ cutoffs used to select candidates for the final array experiment for at least one receptor, and thus could have been discovered using this computational approach. Thus, used together, STATIUM_SC_ plus PSSM_SPOT_ proved effective for identifying new BH3-like peptide binders. At the end of the Results section, we compare the prediction accuracy of all scoring models on our newly compiled data set of binders and non-binders.

We used the solution interaction data of Figure 2 to retrospectively analyze the effectiveness of the SPOT arrays for identifying true interactions. Comparing binding affinities measured in solution to SPOT array signals showed that these were poorly correlated for the diverse set of peptides we tested. This contrasts with previous experiments in which SPOT signals for a series of point mutants of Bim BH3 correlated well with solution binding affinities [19]. In this work, even strong solution interactions sometimes gave very weak signals on the arrays. This was not entirely unexpected, given that synthetic yields probably varied with sequence for this diverse set of peptides. We observed that reduction of the SPOT array signal upon introducing negative control mutations was a better indicator of binding than was the raw signal. For 53 out of 63 solution-validated interactions, both negative control mutations reduced the signal for these interactions on the arrays by at least 30%; for 8 of the 10 exceptions there was a smaller decrease in signal and for 2 there was no detectable change. Several interactions observed on the array but not in solution also passed this cutoff (7 of 28 tested), but it is possible that these “non-binders” do associate with Bcl-2 receptors at concentrations greater than 10 µM.

### SPOT array testing of BH3 motifs from the literature

Among peptides that met the criteria for array IV were several sequences reported in the literature as BH3 motifs but not, to our knowledge, verified to bind as short peptides. We tested several of these interactions on the arrays. Peptides from Mule, Bok, Bcl-g and Bfk had signals ranging from 24% to 250% of the Bim BH3 signal, with Mule and Bok binding specifically to Mcl-1 and not to any of the other four prosurvival proteins on the array (see Data S1). These peptides have functional links to Bcl-2 family biology, and Mule and Bok have been found to interact specifically with Mcl-1 as full-length proteins [25], [26]. When control mutations were introduced into these peptides, the signal was consistently reduced by more than 30%. On the other hand, previously suggested BH3 motifs from CHMP5 (Spike) and RAD9 were also predicted to be binders by our scoring models and were tested on array IV [15]. RAD9 had low signal and modest reduction in signal for the 3aD mutation, which was the only control tested (Data S1). The negative controls for Spike did not reduce the binding signal at all (from a base of 3–6% of the Bim signal; see Data S1). A peptide based on the putative BH3 motif in Aven showed binding to Bcl-x_L_, with an array signal of 11% of the Bim BH3 signal, and >55% reduction in signal for the negative controls on array I; this region of Aven has previously been recognized to have certain features of BH3 motifs [15], and Aven is known to bind to Bcl-x_L_ [27]. Although the STATIUM_SC_ Bcl-x_L_ score for Aven was modest (Z = 0.75), its PSSM_SPOT_ score was better (Z = 3.0). We were not successful in making a soluble peptide based on the Aven BH3 region for solution testing.

Additional putative BH3 motifs postulated based on sequence inspection have been summarized [15], and to our knowledge no peptide binding data exist to validate them as BH3 motifs. Of these sequences, all except APOL1 and APOL6 gave PSSM_SPOT_ and/or STATIUM_SC_ scores worse than the genomic average (i.e. Z-score<0). We tested a peptide from APOL6 for Bcl-x_L_ binding on array II (Z-scores from different models and receptors ranged from 1.8 to 2.7), but at the time did not judge its signal of 17% of the Bim BH3 signal high enough to test with negative controls.

### Diverse binding specificities of newly identified BH3 peptides

The newly identified BH3-like peptides showed a range of binding specificities for the five prosurvival proteins. Peptides from PXT1 and c6orf222 bound all 5 receptors with K_D_<30 nM, but most of the other peptides tested were selective for one or more prosurvival receptors (Figure 2). Three peptides (SNTG2, PCNA, DDX4) bound tightly to Mcl-1, but weakly or undetectably to other receptors, which is a specificity profile similar to known BH3 motifs from BH3-only proteins Noxa and Mule. In agreement with previously reported specificity trends, Bcl-x_L_, Bcl-w and Bcl-2 shared more binding partners in common with each other than with Mcl-1 and Bfl-1 [10]–[14]. Peptides from MCF2L, NBEAL2, POFUT2 and FOXJ2 all bound tightly to Bcl-x_L_, Bcl-w and Bcl-2, but weakly or undetectably to Mcl-1 and Bfl-1, which is a specificity profile previously observed for the BH3 peptide from Bad [10]. We also observed specificity profiles not represented among established BH3 peptides. For example, PURB bound Bcl-2 with a K_D_ value of 40 nM, but bound weakly or undetectably to all other receptors. We used the patterns of binding and non-binding to different prosurvival proteins to test our computational methods, as described below.

### Sequences and structures of novel BH3 motifs

**Figure 3:**
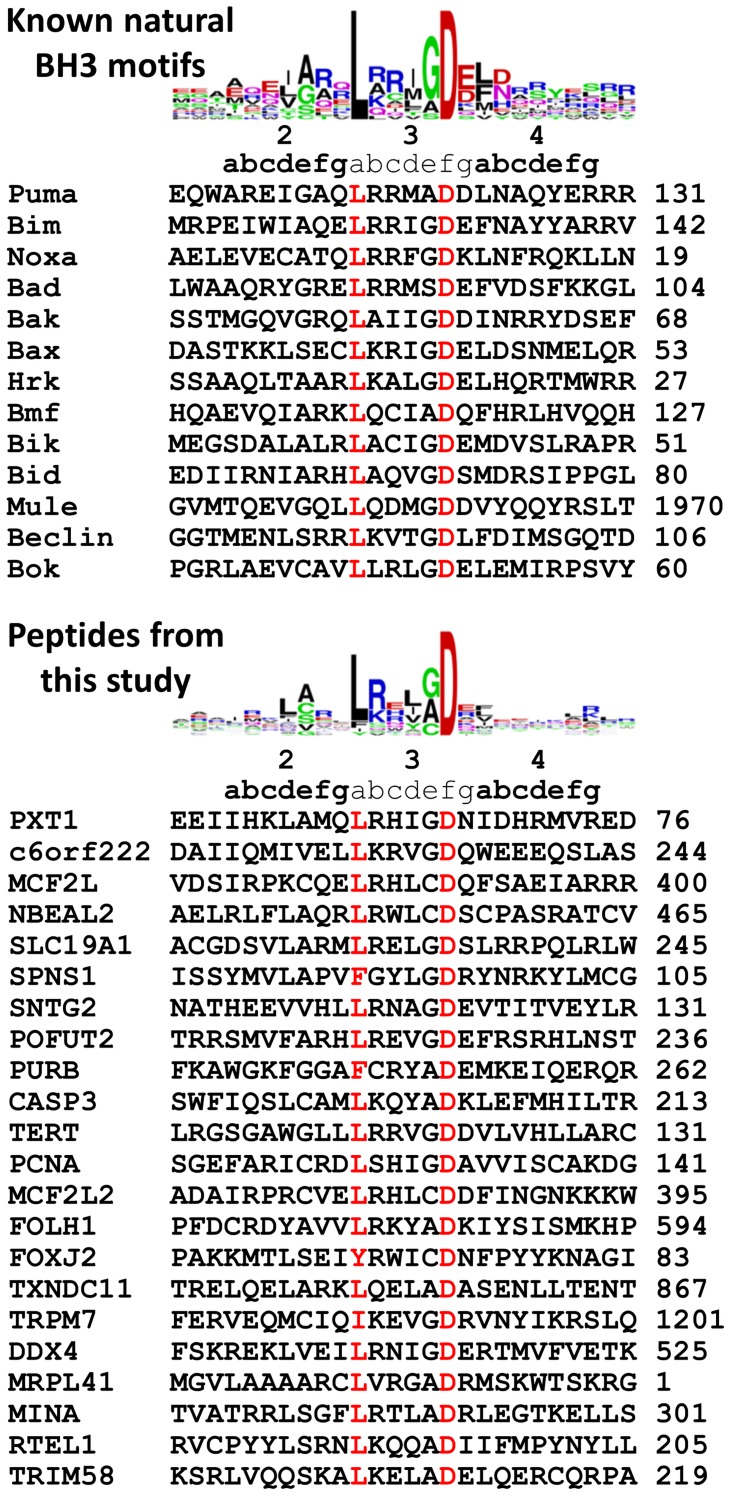
New tight-binding BH3 peptides. Sequence logos and multiple-sequence alignments constructed using BH3 motifs from known BH3-only/pro-apoptotic effector Bcl-2 family proteins or tight binders (K_D_<500 nM) from this study. Highly conserved positions 3a and 3e are colored red. The position of the first residue of the peptide in the full-length protein follows the protein name.

The sequences and sequence logos for tight-binding peptides discovered in this work (K_D_≤500 nM) are compared to those of known natural BH3 motifs in Figure 3. Positions 3a and 3f are highly conserved as leucine and aspartic acid, respectively, in both sets of sequences. There are some exceptions at 3a, however, with Tyr, Phe and Ile found at this position in several of the new peptides. Also, negatively charged residues appear less prevalent at 3g in the logo constructed from newly identified sequences, and more prevalent at 3c. Additional residues are found at 3a and 3f in weaker binders (Table S4). New amino acids were observed at all 26 positions in the multiple-sequence alignment, increasing the sequence diversity of experimentally verified BH3-like peptide binders.

**Figure 4:**
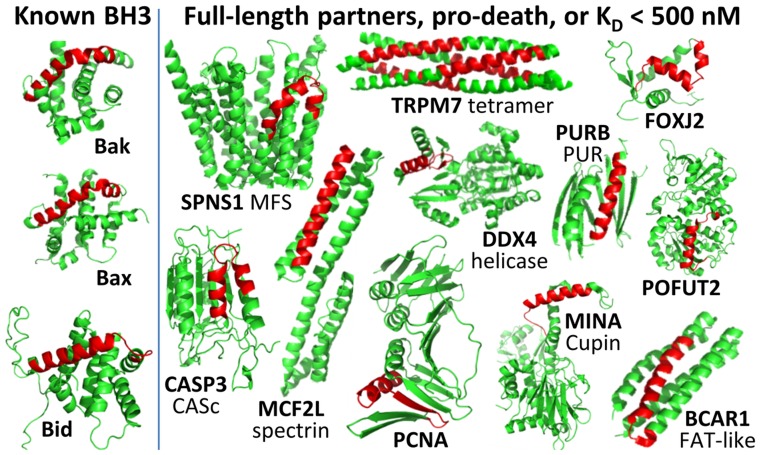
Structures of domains containing known and predicted BH3 peptides. The putative BH3 is shown in red. For Bak, Bax, Bid, POFUT2, TRPM7, PCNA, MINA, DDX4 (*Drosophila*), CASP3 and BCAR1, the structure shown is the structure of the predicted BH3-containing protein. Other BH3 motifs are highlighted in the structure of the closest CDD hit to the parent protein (domain in non-bold type). All PDB IDs are listed in Table S5.

Among known BH3-containing proteins, Bax, Bak and Bid have helical, globular structures that largely bury the hydrophobic residues of the BH3 motif in the protein core [28] (Figure 4). Others such as Bim, Bad and Bmf are intrinsically disordered, and their BH3 motifs adopt a helical conformation upon binding to prosurvival receptors [28], [29], or are predicted to do so (Table S5). We examined the structures available for full-length parent proteins of the 36 peptides that we verified to bind in solution. For 24 proteins, the region containing the BH3 was deposited in the PDB, or there was a conserved domain with a representative structure in the Conserved Domain Database (CDD [30]). Figure 4 highlights residues corresponding to the putative BH3 motifs in these structures in red. The predicted motif is typically a straight or bent helix or, in two cases, a helix-turn-strand. In all cases the motif is integrated into a folded domain. Another 10 putative BH3 regions were predicted to have high-confidence secondary structure in the region of the protein containing the BH3 (Table S5), suggesting that they are also part of folded domains. Thus if any of the peptides we tested do engage with prosurvival proteins as BH3-like helices, almost all would have to undergo a conformational change in order to interact through the predicted motif, as is the case for Bax, Bak and Bid. Two exceptions are the putative BH3 motif in MRPL41, which is located immediately at the N-terminus of (but not in) a single conserved domain for which no structure is available, and C6orf222, which is predicted to be intrinsically disordered. PDB IDs for all structures used in this analysis are included in Table S5.

### Full-length protein-protein interaction survey for Bcl-x_L_ and Mcl-1

For 76 peptides tested on arrays that were also available for expression as the full-length parent protein [31], we tested interaction of the full-length protein with Bcl-x_L_ and Mcl-1 using the LUMIER method [32]. This included 35 proteins that were predicted to contain BH3 motifs but that failed our screening criteria on the SPOT arrays (i.e. gave low signal or failed controls), possibly because they were not completely synthesized. Bim and Bak, which are known to interact with Bcl-x_L_ and Mcl-1 as full-length proteins, were also included [11], [14]. The Bim signal was 7–10 standard deviations higher than the mean of a Gaussian fit to the distribution of signals for binding to Bcl-x_L_ (Z score range of 7–10). For Mcl-1, the Z-scores ranged from Z = 10 to Z = 12 for four replicate experiments (Data S2). Bak binding ranged from Z = 1–3 for Bcl-x_L_ and Z = 6–7 for Mcl-1. EGFP was used as a negative control and had a signal that ranged from Z = −1.5 to Z = 0.0 for Bcl-x_L_ and Z = −1.6 to Z = −0.3 for Mcl-1. Of our validated peptides, c6orf222 had the highest signal for Bcl-x_L_ binding, which ranged from Z = 5 to Z = 9 for four replicates. For other proteins, the replicate values were too noisy to confidently assign any hits, although there were some indications of weak binding. Weak hits included CHMP5 (Spike) and Aven, proteins with putative BH3 motifs identified through sequence inspection [15]. Complete LUMIER data are included in Data S2.

### Assessing binding prediction performance

To assess the predictive accuracy of our models using the new peptide binding data, we compiled a list of 128 peptides for which 412 interactions have been measured for binding to 2–5 receptors (Methods and Data S3). It should be noted that all peptides in this test set have properties of BH3 motifs, and all but one have been demonstrated to interact with at least one prosurvival protein (Data S3). In two different tests, we compared the predictions of four models: STATIUM, STATIUM_SC_, PSSM_SPOT_ and PSSM_SPOT_+STATIUM_SC_ (the PSSM_SPOT_+STATIUM_SC_ score is the average of the Z-scores for these two models, see Methods).

**Figure 5:**
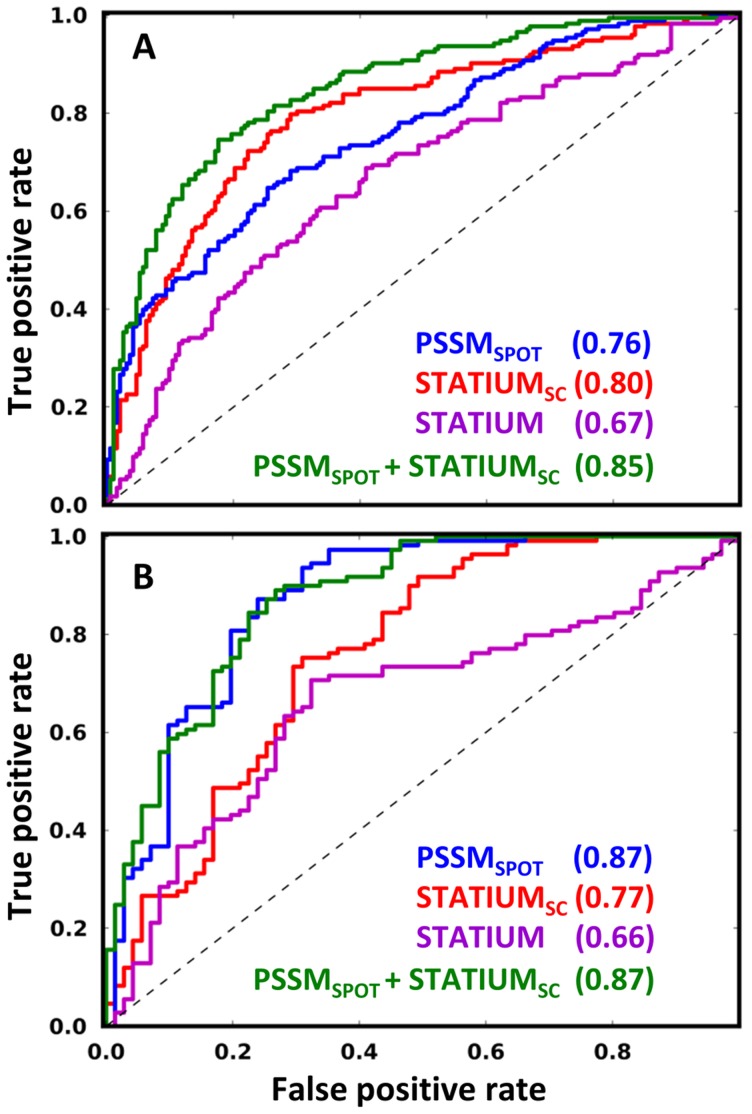
Predicting peptide binding to the 5 Bcl-2 receptors. The first benchmark (A) included 366 interactions (K_D_<1 µM) and non-interactions (K_D_>10 µM). Four models were evaluated with respect to their ability to correctly classify each example, as a function of the score cutoff used for prediction. The second benchmark (B) included 180 comparisons of one receptor binding a peptide (K_D_<1 µM) and another receptor not binding that same peptide (K_D_>10 µM). The difference in scores for a peptide binding to two receptors was used to predict the binding preference, and agreement with experiment was evaluated as a function of the score difference cutoff. The “PSSM_SPOT_+STATIUM_SC_” score is the average of the Z-scores of the two models for a given receptor. Values in parentheses report the area under the curve (AUC) for each method. For details, see the Methods section.

Figure 5 shows ROC curves of the true positive rate vs. false positive rate for predicting strong vs. non-interactions in the test set. All models had predictive capability as assessed using the area under the ROC curve (AUC): the 90% confidence intervals for predicting binding were 0.72–0.80 for PSSM_SPOT_, 0.76–0.84 for STATIUM_SC_, 0.62–0.71 for STATIUM, and 0.82–0.88 for PSSM_SPOT_+STATIUM_SC_ (Figure 5A). STATIUM_SC_ outperformed STATIUM (AUC = 0.80 vs. 0.67) and the combined PSSM_SPOT_+STATIUM_SC_ outperformed its individual components (AUC = 0.85). When the Bim variants were removed from the test set (because these might be easier to predict, given that the PSSM_SPOT_ model is based on mutations made in Bim BH3), the AUC values remained similar: 0.72 for PSSM_SPOT_, 0.75 for STATIUM_SC_, 0.62 for STATIUM, and 0.80 for PSSM_SPOT_+STATIUM_SC_.

To assess the ability of our models to predict the binding preferences of different receptors, we identified a subset of peptides for which experiments support binding to one receptor (K_D_<1 µM) but not to an alternative receptor (K_D_>10 µM). We used our four models to predict these binding preferences (see Methods). As shown in Figure 5B, the AUC values for this test were generally higher than those for predicting binding vs. non-binding. We observed this result previously when predicting affinity and specificity for SPOT array data [19]. 90% confidence intervals for specificity prediction are 0.82–0.91 for PSSM_SPOT_, 0.69–0.82 for STATIUM_SC_, 0.59–0.73 for STATIUM, and 0.82–0.91 for PSSM_SPOT_+STATIUM_SC_.

The analysis of prediction performance summarized in Figure 5 is sufficient to generate statistically robust conclusions about model performance. On the other hand, it can mask differences in performance that vary by receptor. For example, restricting specificity prediction to the 85 peptides that are specific for binding to Bcl-x_L_ over Mcl-1, or vice versa, results in nearly perfect prediction for all models (0.99 for PSSM_SPOT_, 0.96 for STATIUM_SC_, 0.99 for STATIUM, and 1.0 for PSSM_SPOT_+STATIUM_SC_). This result holds whether the analysis is restricted to the Bim BH3 variants or the more diverse natural peptides.

## Discussion

Our goal in this work was to use models that capture determinants of BH3 peptide binding to discover new candidate BH3 motifs in the human proteome. We identified 34 peptides that bound at least one Bcl-2 receptor with K_D_≤5 µM and 22 peptides that bound with K_D_<500 nM (Figure 2). Some of these peptides have novel specificity profiles for binding to human prosurvival Bcl-2 proteins, which could provide insights into their possible functions or make them useful as reagents [10]. We used our large amount of new experimental data to compare the prediction accuracy of different models and investigate properties of the predicted binders.

Whether any of the BH3-like peptides that we identified here participate in functionally relevant interactions with prosurvival Bcl-2 receptors in cells can only be resolved by further investigation of individual targets, which is beyond the scope of this work. However, we searched the literature for functional information about our newly identified BH3-like proteins and identified reports that link some of the proteins to cell-death biology. For example, several tight-binding BH3-like peptides reported here (all with K_D_<500 nM) are found in proteins previously observed to interact directly with Bcl-2 receptors as full-length proteins: PCNA, CASP3, SPNS1 and MRPL41. CASP3, or caspase 3, cleaves Bcl-2 to produce a Bax-like death effector [33]. SPNS1, or HSpin1, is localized to mitochondria, and SPNS1 expression induces caspase-independent necrotic cell death that can be blocked by Bcl-x_L_ [34]. PCNA, proliferating cell nuclear antigen, is a processivity factor for DNA polymerase δ that is involved in DNA replication and repair. PCNA has been reported to bind Mcl-1 and mutations in Mcl-1 near the BH3-binding groove disrupt the interaction with PCNA [35]. Interactions with full-length PCNA, CASP3 and SPNS1 may be mediated by structures distinct from the BH3 regions we predict; e.g. a deletion test supports an alternative mode for SPNS1 [34]. For MRPL41, on the other hand, yeast two-hybrid data support an interaction mediated by our predicted BH3 region. MRPL41 (also known as BMRP) is an apoptosis-inducing mitochondrial protein that interacts with Bcl-2. Mutation of Asp16 to Ala in full-length MRPL41 abolished Bcl-2 binding in a two-hybrid assay [36]. This residue corresponds to the conserved aspartate in our predicted BH3 motif, which as a peptide binds Bcl-2 with a K_D_ of 190 nM. Interestingly, the Asp to Ala mutation did not diminish apoptosis induced by MRPL41 overexpression. Conde et al. speculate that Bcl-2 binding may modulate a pro-apoptotic function of MRPL41 [36]. In full-length MRPL41, the putative BH3 motif is located at the N-terminus and is the only part of the protein not predicted to lie in a conserved domain. The cell death regulator Aven, which participated in candidate array interactions with Bcl-x_L_, has also been reported to bind as a full-length protein to Bcl-x_L_, and it has been noted before that the region we predicted has BH3-like characteristics [27]. Over-expression of MCF2L (alias Ost), which is a Rho GTPase exchange factor, induced fibroblast cell death upon serum withdrawal, with DNA fragmentation characteristic of apoptosis [37].

Other peptides identified in this work have functional connections to Bcl-2 family biology and/or apoptosis. PXT1, which binds tightly to all five prosurvival proteins (Figure 2), is a peroxisomal protein that promotes apoptosis in sperm cells. The BH3 motif we predict was recognized by Kaczmarek et al., who demonstrated that deleting this region in PXT1 significantly reduces PXT1-induced apoptosis [38]. The channel protein TRPM7 has pro-apoptotic function mediated by caspases, which cleave the kinase domain of TRPM7 from its transmembrane channel domain. This activity controls the participation of TRPM7 in Fas-induced apoptosis [39]. The BH3-like motif we predict in TRPM7 is located in the cytoplasmic coiled-coil tetramerization domain, which is included with the channel domain in the cleavage product [40] (Figure 4). BCAR1 is reported to have a pro-apoptotic function when cleaved by caspase 3 [41]. The C-terminal cleavage product includes the sequence fragment corresponding to the FAT-like domain shown in Figure 4, which contains a sequence motif that we determined to be Mcl-1-specific as a peptide in solution. Over-expression of the C-terminal domain of BCAR1 leads to caspase-dependent cell death.

In addition to assaying peptide interactions, we tested full-length target proteins for association with Bcl-x_L_ and Mcl-1. Binding to a putative BH3 region in a full-length protein depends on its accessibility. The LUMIER assay tests prey with unknown folding status in a 293T cell line. We noted that both Bim BH3 and c6orf222 (which bound to Bcl-x_L_ and Mcl-1 with K_D_<30 nM as peptides) gave strong signals in the LUMIER Bcl-x_L_ assay when tested as full-length proteins. Bim is intrinsically disordered and C6orf222 is predicted to be, which would make their BH3 motifs accessible for interaction. Many of the other predicted BH3 motifs that we identified are predicted to lie in structured domains, as illustrated in Figure 4, and may require conformational changes, protease cleavage or cellular chaperones to regulate binding. Interestingly, SPNS1 was observed to interact with Bcl-x_L_ and Bcl-2 in an immunoprecipitation assay only after treatment of cells with pro-apoptotic stimuli, suggesting that a conformational change in this protein is required for interaction [34]. Thus, it is not surprising that we do not see more strong interaction signals when testing full-length proteins in the LUMIER assay.

When comparing our models with respect to their prediction performance discriminating binders vs. non-binders, the highest accuracy resulted when data-based PSSM_SPOT_ and structure-based STATIUM_SC_ models were used together (Figure 5). This result is not surprising, considering that in our genome search we found that using those two scoring models together resulted in the greatest enrichment of predictions in candidate array interactions. Each model has strengths and limitations. PSSM_SPOT_ is derived from experimental binding data collected for Bim BH3 mutants, but measurements are currently available for only a subset of peptide positions. PSSM_SPOT_ also makes the strong assumption that residue contributions are fully independent and do not vary with sequence context. STATIUM_SC_ can score all amino acids at all positions, but is limited in our current implementation by scoring all binding to a given receptor protein using a single structural scaffold; STATIUM_SC_ also assumes that contributions from different sites are independent. Strikingly, STATIUM_SC_ performed as well as PSSM_SPOT_ on the large-scale binding prediction test in this paper without using any Bcl-2-specific information other than the input structures (Figure 5).

PSSM_SPOT_ and STATIUM_SC_ models are sufficiently predictive to identify many BH3-like sequences in the human proteome that bind as peptides to prosurvival proteins. It is interesting to consider whether additional BH3-like binders remain to be discovered. We think this is likely. Most immediately, 51 peptides that participated in candidate array interactions in this study were not tested in solution. Of these, 28 had PSSM_SPOT_+STATIUM_SC_ scores greater than Z = 2.0. Of candidate array peptides that met this Z-score cutoff and were tested in solution, 22 out of 34 bound with K_D_<1 µM to at least one prosurvival protein. Thus, these 28 candidates could be prioritized based on their scores or functional annotation for further testing. Beyond candidate array interactions that we have already identified, there may be other authentic BH3 sequences in the proteome that do not score well with our models. As discussed above, PSSM_SPOT_ and STATIUM_SC_ each make strong assumptions that could introduce biases into the predictions. Figure 3 shows that new sequences that we identified included residues not found in previously known BH3 motifs, at every BH3 position. But overall, the sequences of new and previously known binders share similar characteristics. This is expected, given that both the PSSM_SPOT_ and STATIUM_SC_ models were constructed using mutational data or structures from known complexes. If less canonical BH3 peptides exist in the proteome that have divergent sequences, and/or that bind with altered geometry, these would likely go undetected by our current methods.

Although the PSSM_SPOT_ model can only be used to predict Bcl-2/BH3 interactions, the enhanced version of STATIUM that we present here, STATIUM_SC_, can be applied to any of thousands of protein-protein interactions for which a structure is available. We are testing and optimizing STATIUM_SC_ for more general application. Due to its ability to rank interactions among structurally similar human paralogs, STATIUM_SC_ could serve as a fast and inexpensive tool to enhance other computation-driven efforts to elucidate the human protein-protein interactome [42].

## Methods

### PSSM_SPOT_ and STATIUM models

The PSSM_SPOT_ models have been described and were used as reported by DeBartolo et al. These models were derived from experiments performed for each Bcl-2 family protein receptor [19]–[21]. Briefly, a residue at a given site in a BH3 peptide was assigned a score determined by the signal for binding to the receptor on a SPOT array when that substitution was made in the context of Bim BH3 (signal intensity I_SPOT_), normalized by the Bim BH3 signal: S_SPOT_ = −(log(I_SPOT_)−log(I_SPOT_BIM_)).

STATIUM and STATIUM_SC_ scoring models are derived from a template protein structure and are used to score the compatibility of a sequence with the structure of the template; the structures used in this work are specified below. The original STATIUM model considered any residue pair in the template structure with Cβ atoms less than 10 Å apart and with Cα-Cβ vectors not pointed away from each other to be interacting [19]. For STATIUM_SC_, which we have applied so far only to protein-peptide complexes, if any atom in a receptor sidechain is within 6 Å of an atom in a peptide sidechain, the residue pair is considered to be interacting. Given a list of interacting residue pairs in the template, the next step is to identify structurally similar interacting pairs in a large database of known structures. For this purpose we compiled a subset of PDB structures, as described in DeBartolo et al. [19]. The subset consists of 19384 non-redundant, high-resolution and high-quality single-chain structures, with no restrictions on the type of protein or organism of origin. We used the same interaction criterion applied for the template to identify interacting pairs in the PDB-derived database of structures. In order to compare an interacting residue pair from the template to one from the PDB, all distances between pairs of atoms, one in residue_i_peptide_ and one in residue_j_receptor_, are calculated, where residue_i_peptide_ is the residue at position i in the peptide and residue_j_receptor_ is the residue at position j in the receptor. For this step, only the C_α_ and C_β_ atoms of residue_i_peptide_ are considered. The list of PDB interacting pairs is then searched to find cases where the amino-acid identity of one member of the pair is identical to that of residue_j_receptor_; residues in a PDB pair are referred to as residue_j_PDBreceptor_ and residue_i_PDBpeptide_. To evaluate the structural match between a residue pair in the template of interest and a PDB residue pair, all non-hydrogen side-chain atoms are considered for residue_j_PDBreceptor_, but only the C_α_ and C_β_ atoms are considered for residue_i_PDBpeptide_. The distances between atoms in residue_i_PDBpeptide_ and residue_j_PDBreceptor_ are then calculated and compared to equivalent distances in residue_i_peptide_ and residue_j_receptor_. If the root-mean squared difference of all distances is less than 0.4 Å, the pairs are considered structurally similar and the amino-acid identity of residue_i_PDBpeptide_ is added to the count matrix of that pair. The 0.4 Å distance cutoff was determined by decreasing a starting cutoff of 1.0 Å in units of 0.1 Å. We found that the Z-scores of known binders increased for all receptor models as the cutoff was decreased up until 0.4 Å. At that cutoff, several pairs had fewer than 100 counts, suggesting that below that cutoff there was insufficient data in the PDB to derive the potential. The final contribution of residue_i_peptide_ to the STATIUM_SC_ score is given by Σ(−log(P_AAi_/P_AAPDB_)), where *P_AAi_* is the frequency of the amino acid (AA) at peptide position *i* (residue_i_peptide_) in matching pairs and *P_AAPDB_* is the frequency for residue_i_peptide_ in the culled PDB.

### Structure templates

The crystal structures used to generate STATIUM_SC_ models were the same as those used to generate STATIUM models in our previous study: Bcl-x_L_:3io8 (Bcl-x_L_ bound to Bim3aF BH3 [22]), Mcl-1:3pk1 (Mcl-1 bound to Bax BH3 [23]), Bfl-1:3mqp (Bfl-1 bound to Noxa BH3), Bcl-2:2xa0 (Bcl-2 bound to BaxBH3 [43]) [20]. For Bcl-w, two models were considered. One was PDB entry 1zy3, which is a model based on NMR data [44]. We also generated a homology model using SCWRL4 based on the crystal structure of Bcl-2 (2xa0), which is most similar in sequence to Bcl-w (46% sequence identity). The Cartesian coordinates of identical residues were fixed to those of Bcl-2 and served as steric boundaries for Bcl-w sidechains [45]. The resulting model had slightly better average Z-scores for established Bcl-w-binding BH3 motifs, so we reported prediction results using that.

### Scoring candidate BH3 motifs in the human proteome

Starting with 30,046 sequences in the Human Protein Reference Database [46] as of 07/20/10, we generated a list of 591,829 26-residue sequences as follows. To approximate the compositional profile of known BH3 motifs, sequences were required to include at least 35% polar/charged residues (DEHKNQRST). Also, at most one proline was allowed between positions 2d and 4a. A nonpolar amino acid at position 3a (FILVYWM) and a small residue at position 3e (ACGS) were also required. The constraints at these two positions made it easier to construct a non-redundant set of sequences for testing. After filtering, each of the candidate sequence windows was scored with each of 15 models (PSSM_SPOT_, STATIUM and STATIUM_SC_ models for each of 5 prosurvival proteins).

### SPOT array experiments

Peptide SPOT arrays were synthesized using F-moc chemistry on nitrocellulose membranes at the MIT Biopolymers facility using an Intavis AutoSpot robot. Peptide spots were cut from the membrane, hydrated in 100% methanol, transferred to TBS (50 mM Tris, pH 8.1, 100 mM NaCl, 0.01% Triton X-100) with 1% bovine serum albumin (BSA), here called blocking buffer, and incubated at room temperature for ∼10 minutes. Membranes were then incubated with 10 ml of 1 µM or 100 nM c-Myc-tagged receptor (sequences in Table S6) in TBS for 1 h at room temperature. Membranes were then rinsed 3× with blocking buffer and then incubated with anti-c-*myc*-Cy3 antibody (Sigma Aldrich C6594) diluted 100-fold in blocking buffer for 1 hour at room temperature. Membranes were rinsed 3× with blocking buffer and scanned on a Typhoon 9400 (GE Healthcare). Images were analyzed with ImageQuant (GE Healthcare), and Cy3 intensity at 580 nm was averaged over a circular area that was equal in size for all spots for a given membrane. Typically, 5–10 known binders and their negative controls were included.

### Selection of peptides for SPOT array testing

When selecting candidate peptides to be tested on SPOT arrays, lists of peptides resulting from computational analysis were visually inspected for long stretches of hydrophobic amino acids that might affect solubility. Additionally, sequences with stretches of like-charged amino acids or glycines were excluded. For sequences that were more than 75% similar, we used the best-scoring sequence rather than testing both.

For array I, 127 peptides were tested for binding to all five receptors (Table S2). This array featured a broad range of predictions designed to narrow down potential follow-up array experiments. The top scoring peptides according to each of our receptor models (5 models each for PSSM_SPOT_ and STATIUM) were synthesized and tested on the array, but there was significant overlap in the list. For example, 96 of the 127 peptides had PSSM_SPOT_ Z-scores better than 2.0 for at least one receptor, and 28 had Z>2.0 for all 5 receptors (82 passed for 2 or more receptors). Peptides with Z>2.0 based on PSSM_SPOT_ were evenly distributed across the five receptors (72 for Bcl-x_L_, 61 for Bcl-w, 63 for Bcl-2, 62 for Mcl-1 and 61 for Bfl-1). 29 peptides had Z>2.0 based on STATIUM (13 for Bcl-x_L_, 2 for Bcl-w, 21 for Bcl-2, 12 for Mcl-1 and 10 for Bfl-1), of which 13 only passed the threshold for one receptor. In addition to the genome-wide top scorers, we also tested the top-scoring peptides within known Bcl-2 family receptor interaction partners Aven, CASP8, CASP5, BAR, and Tankyrase, even though these were not top scorers genome-wide. We also probed BH3 sequence diversity by prioritizing peptides with non-canonical substitutions at positions 3a and 3f, which are conserved as Leu and Asp in established motifs.

Array II included the top-scoring 176 peptides according to Bcl-x_L_ STATIUM, which were tested only for Bcl-x_L_ binding, and the top-scoring 175 peptides according to Mcl-1 STATIUM, which were tested only for Mcl-1 binding. Peptides already tested on array I were not included. Negative controls were only tested for a subset of array II interactions with the highest signal, and no peptides from array II were tested in solution.

Array III peptides were selected from 1823 proteins reported to interact directly with Bcl-2 receptors, or with proteins that interacted with Bcl-2 receptors (2 degrees of separation) according to the Human Protein Reference Database [46]. The top-scoring peptides in this set according to STATIUM that also had PSSM_SPOT_ Z-scores better than 3.0 were selected. We also required that putative BH3 regions were more conserved than the rest of the protein in which they were found, which was observed in the full-length proteins for established peptide binders Bim, Bad, Bik, Bid, Bmf, Hrk and Noxa. To do this, we used default NCBI Blastp to generate multiple sequence alignments (MSA) of full-length mammalian proteins. We calculated the Shannon entropy, S = (−Σ(P*log(P))), for 12-residue windows across the MSA. We used a 12 residue window because the sequences of known BH3 domains are most conserved between positions 2d through 4a. In order to be considered conserved, the entropy of the 2d-4a window of the candidate peptide had to be lower than the average of all other windows in the MSA. On array III we also included 14 peptides with Glu at position 3f that had PSSM_SPOT_ Z-scores better than 2.5, under the hypothesis that the strong preference of PSSM_SPOT_ for Asp at position 3f is introduced because of the Bim sequence context of that model. These peptides were not subjected to the constraints described above; they were selected using PSSM_SPOT_ alone.

For array IV, we only predicted and tested interactions with Bcl-x_L_, Mcl-1 and Bfl-1. We used a combination of PSSM_SPOT_ and STATIUM_SC_ scores to select candidates for testing. We set a PSSM_SPOT_ Z-score cutoff that was the score of the worst scoring known BH3 peptide binder: Bcl-x_L_/Beclin BH3 Z = 3.2; Mcl-1/Puma BH3 Z = 3.4; Bfl-1/Puma BH3 Z = 3.7. We then took the top 20 STATIUM_SC_-scored peptides over that threshold. We also required that the STATIUM_SC_ score be less than 0.0, so for some receptors we tested fewer than 20 candidates. We then repeated the analysis, reducing the PSSM_SPOT_ cutoff by one unit of raw score, to generate more candidate peptides for testing. The corresponding new Z-scores were as follows: Bcl-x_L_, Beclin Z = 2.2; Mcl-1, Puma Z = 3.2; Bfl-1, Puma Z = 2.0. 107 peptides were tested on array IV for binding to Bcl-x_L_, Mcl-1 and Bfl-1. Predictions were made for Bcl-x_L_, Mcl-1 and Bfl-1 in that order (e.g. a peptide predicted to bind to Bcl-x_L_ was excluded from the Mcl-1 list, even though it could have had a good Mcl-1 score). Each predicted peptide was tested for binding to all three receptors, even though peptides were selected based on their scores for only one of the five receptors.

### Fluorescence polarization binding assays

Peptides were synthesized with an N-terminal fluorescein group (FAM) and an amidated C-terminus; mass spectrometry confirmed the correct mass in the crude sample. Peptides were purified by reverse-phase HPLC using a C18 column and a linear water/acetonitrile gradient. We re-analyzed the purified peptide by mass spectrometry if there was not a single well-defined peak in HPLC chromatogram. In all cases reported here, the purified samples that were re-analyzed contained the correct mass.

Fluorescence polarization (FP) assays were done at 25°C in assay buffer (20 mM NaPO_4_, 50 mM NaCl, 1 mM EDTA, 0.001% Triton X (v/v), pH 7.8). For direct binding assays, FAM-labeled peptides were at a concentration of 10 nM, and Bcl-x_L_ or Mcl-1 was serially diluted in 96 well plates. For competition binding assays, the concentration of labeled test peptides was 10 nM, and receptor was fixed at a concentration that gave sufficiently high bound signal (concentrations are given in the figure legends). Unlabeled Bim BH3 was serially diluted in 96-well plates. For all FP experiments, the total well volume was 120 µl. The plates were mixed by shaking at 25°C for 1 hour before the first measurement, and were measured again after 24 hours with no significant change. Anisotropy was measured using a Spectramax M5 plate reader from Molecular Devices.

Direct binding curves were fit using Pylab/Scipy/Optimize to a single-site binding model utilized previously [18]. For SPNS1 binding there was a large change in the raw fluorescence signal upon binding that saturated at high concentrations of receptor, so we fit the raw fluorescence change rather than the anisotropy. The SPNS1 anisotropy data were noisy, particularly for Bfl-1, which was possibly due to the large change in raw fluorescence signal upon binding. For other binding experiments that gave a significant change in fluorescence intensity, fitting the change in anisotropy and the change in fluorescence intensity gave K_D_ values that were the same within ±10%. Presumably due to variation in the mobility of the fluorescent dye when bound to different receptors, the upper baseline anisotropy varied among the five receptors. The 95% confidence intervals for the K_D_ values were used to assess affinity; for weak interactions where the upper baseline was highly uncertain these intervals could be large (see Table S3).

### Assessing the structure of putative BH3-containing proteins

Structures of proteins containing predicted BH3 motifs were obtained from the PDB. When no structure was available, we searched the conserved domain database (CDD [30]) to determine if the region containing the peptide was part of a conserved domain for which a representative structure had been solved. For proteins for which no structural information was available, we predicted secondary structure with PSIPRED [47]. If the PSIPRED prediction was all coil, we predicted intrinsic disorder with DisProt and DisEMBL [48], [49]. Only c6orf222 had coil predicted by PSIPRED and intrinsic disorder by DisProt and DisEMBL throughout its sequence.

### LUMIER

The LUMIER assay was carried out as previously described [32] with the following modifications. A stable cell line expressing receptor tagged on the N-terminus with Nanoluc luciferase [50] was created (the prey). The sequences for the Nanoluc-Bcl-x_L_ and Nanoluc-Mcl-1 constructs are included in Table S6. 79 potential Bcl-x_L_/Mcl-1 binders (the bait) were obtained from the Orfeome collection and cloned into expression vectors with 3xFLAG-V5 appended on the C-terminus [31]. Each bait construct was transfected into the receptor-luciferase cells in a 96-well format and after 48 hours the cells were lysed and transferred to 384-well plates coated with anti-FLAG. After incubation and washing, luminescence was measured to quantify how much receptor-luciferase was captured by the bait protein. Anti-Flag ELISA signal was used to exclude bait proteins that were not expressed. The experiment was carried out two times, each in duplicate. We found that for the second experiment the duplicate values were more correlated to each other than in the first experiment for both Mcl-1 (R1 = 0.83; R2 = 0.96) and Bcl-x_L_ (R1 = 0.41; R2 = 0.6), although the control signals and test hits reported here were consistent across both experiments.

### Binding prediction

We compiled a list of 128 peptides for which 412 interactions have been measured involving binding to 2–5 receptors (Data S3). The test set included 36 newly characterized peptides from this study, 10 natural BH3 domains, a non-binding Bid mutant [10], and 81 Bim variants with multiple point mutations from previous studies [18], [20]. In two different tests, we compared the predictions of four models: STATIUM, STATIUM_SC_, PSSM_SPOT_ and PSSM_SPOT_+STATIUM_SC_. All scores were scaled by calculating the Z-score relative to a dataset of ∼600,000 genomic peptides (see Methods). To combine the PSSM_SPOT_ and STATIUM_SC_ models, we used the average of the Z-scores for each model corresponding to the same receptor.

We examined the ability of different models to distinguish strong interactions from non-interactions by generating ROC curves reporting the true positive rate vs. false positive rate as a function of the score cutoff for predicting an interaction. Comparisons of K_D_ values between studies are complicated by varied peptide lengths and assay conditions. We tested different upper limits for defining a strong interaction (K_D_ = 500 nM, 1 µM or 5 µM). Prediction performance was almost identical for cutoffs of 500 nM vs. 1 µM, but was slightly worse for 5 µM. We reported results using a 1 µM cutoff, defining non-interactions as those protein/peptide pairs with a K_D_ value greater than 10 µM (giving 193 interactions and 173 non-interactions).

In a second test, we examined the scores for a subset of specific binders for which experiments support a peptide binding to one receptor (K_D_<1 µM) but not to an alternative receptor (K_D_>10 µM). As an example of a prediction, a peptide that bound Mcl-1 but not Bcl-x_L_ would be classified as a specific binder. Our dataset included 180 examples of such comparisons, including 99 unique peptides and 5 receptors. We used our models to predict preferential binding by defining a specificity score corresponding to the difference between Z-scores for a peptide binding to two prosurvival proteins. True positive and false positive rates for correctly predicting the binding specificity were computed as a function of specificity score cutoffs.

To compute confidence intervals for the AUC values, we used the bootstrapping protocol described by DeBartolo et al. [19]. Briefly, for each dataset we re-sampled the data 2000 times with replacement to generate the bootstrap distribution. In Figure 5 we report the limits of the 90% confidence interval resulting from this procedure. For ROC curves, the true positive rate is TP/(TP+FN) and the false positive rate is FP/(FP+TN).

## Supporting Information

Figure S1 **Direct binding of peptides corresponding to predicted BH3 motifs to five human Bcl-2 receptors in solution.** Bcl-x_L_ (red), Mcl-1 (blue), Bcl-w (green), Bfl-1 (purple) or Bcl-2 (magenta) were titrated into fluorescein-labeled peptides at a constant concentration of 10 nM. Points are the mean of replicates, and error bars are ±1 standard deviation from the mean of replicates, for illustrative purposes. Curves without error bars are representative curves for cases in which replicates were measured using different concentrations of receptor protein. The K_D_ values and confidence intervals reported in Table S3 resulted from fitting all replicate measurements together.
(PDF)

Figure S2 **Competitive binding of predicted peptides with unlabeled Bim in solution.** Fluorescein-labeled peptides were present at a concentration of 10 nM and the receptor concentration varied depending on the strength of binding. Unlabeled Bim BH3 was titrated. The concentration of receptor was 100 nM for PXT1, 25 nM for MCF2L and NBEAL2, 250 nM for SLC19A1, SNTG2 and POFUT2, 2000 nM for PCNA, 250 nM for FOXJ2 and DDX4, 1000 nM for TERT and CASP3, 3000 nM for MCF2L2, 875 nM for TRPM7 and MINA, 3000 for PLEKHH1 and SPNS1, 930 nM for VCAM1, 875 nM for RTEL1, 875 nM for NUB1, 300 nM for c6orf222, 1 µM for TXNDC11, 1 µM for PURB, 1 µM for FOLH1, 1 µM for TRIM58, 1 µM for ARHGAP and 548 nM for BCAR1. MRPL41 was unlabeled and was used to compete with binding of 10 nM labeled Bim to 50 nM Bcl-x_L_.
(PDF)

Table S1 **Conservation of sidechain structure in Bcl-2 complexes.**
(DOCX)

Table S2 **SPOT array experiments.**
(DOCX)

Table S3 **Best fit K_D_ values and 95% confidence intervals.**
(DOCX)

Table S4 **Sequences of weak peptide binders.**
(DOCX)

Table S5 **Summary of structures of known and predicted BH3 motifs.**
(DOCX)

Table S6 **Sequences of prosurvival receptor constructs.**
(DOCX)

Data S1 **SPOT array signals for Arrays I–IV.**
(XLSX)

Data S2 **LUMIER results of Mcl-1 and Bcl-x_L_.**
(XLSX)

Data S3 **Interaction data used for the prediction benchmarks.**
(XLSX)

Text S1 **Conservation of sidechain structure in Bcl-2 complexes.**
(DOCX)
