# Supplementary figures and images for: Genome-Wide Prediction and Validation of Peptides That Bind Human Prosurvival Bcl-2 Proteins
Source: PLoS Comput Biol. 2014 Jun 26;10(6):e1003693. doi: 10.1371/journal.pcbi.1003693 (PMC4072508; doi:10.1371/journal.pcbi.1003693)

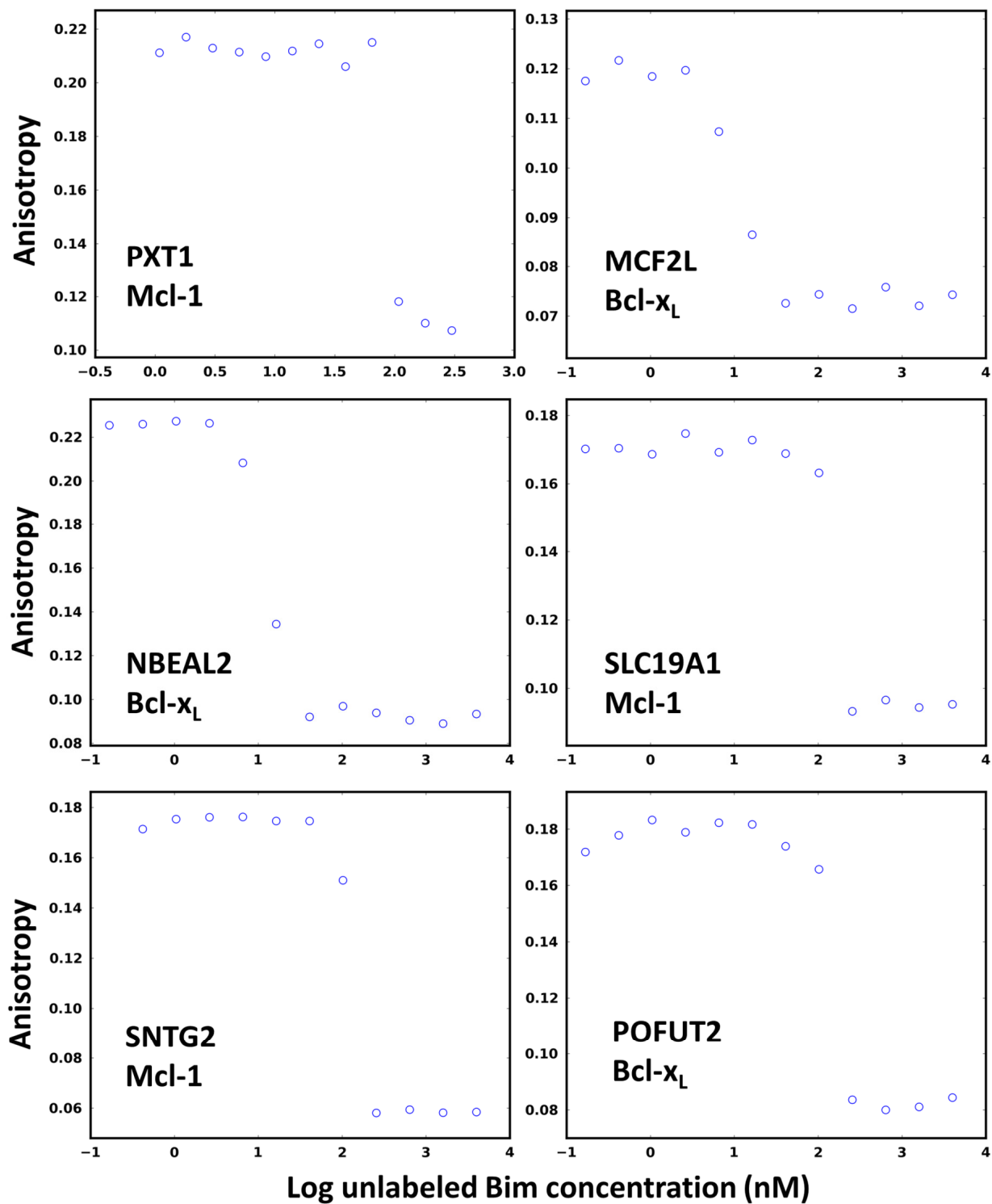

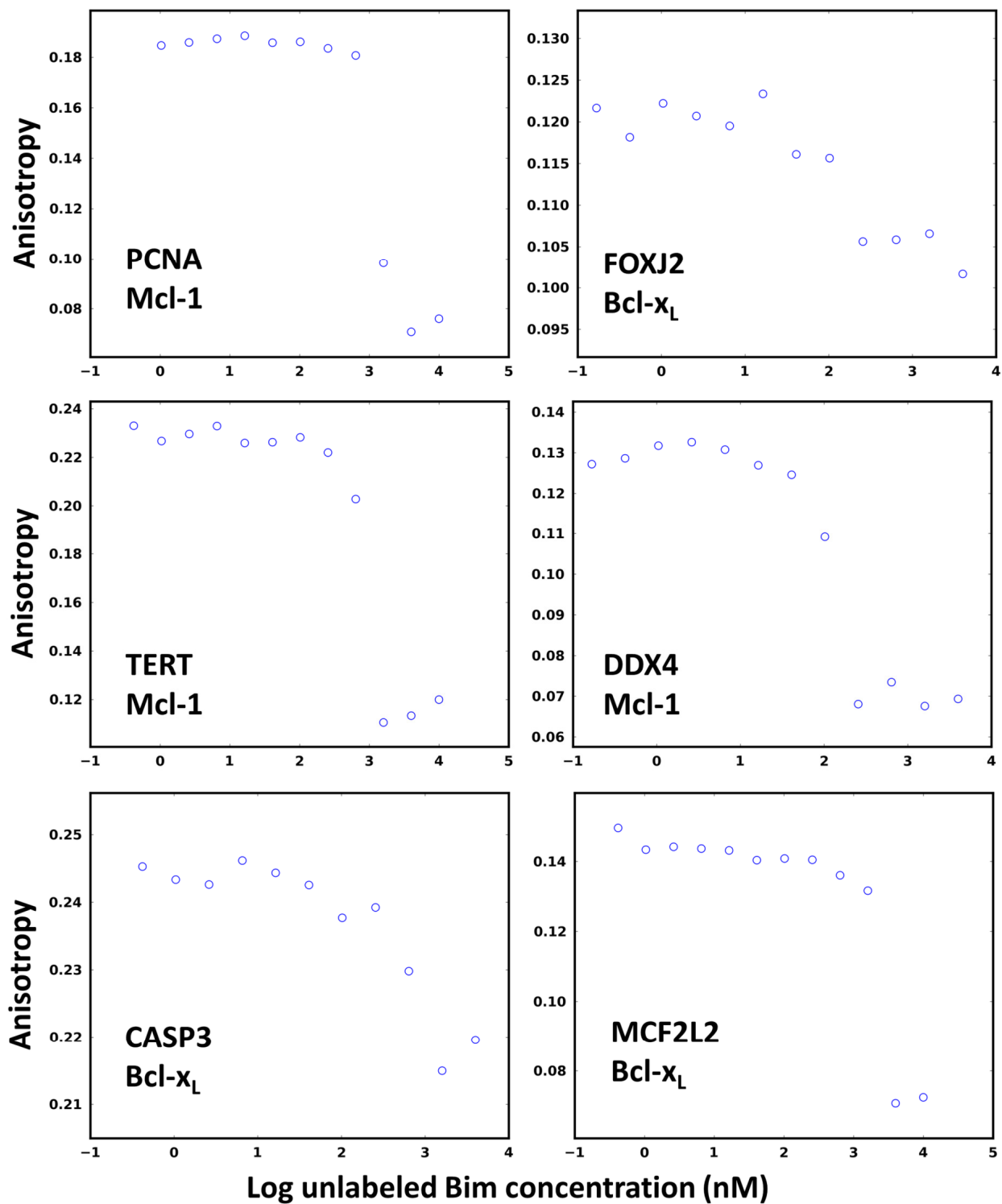

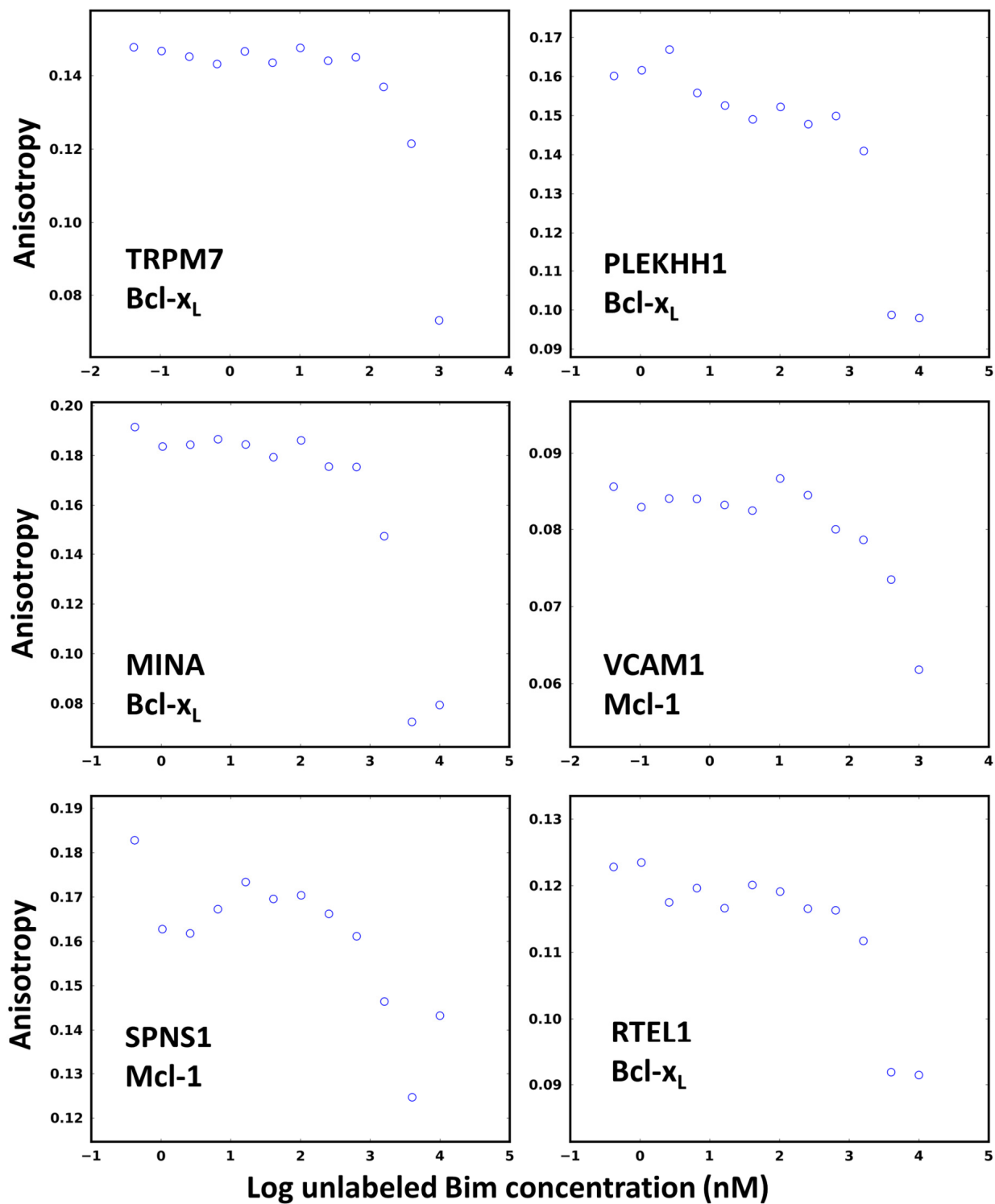

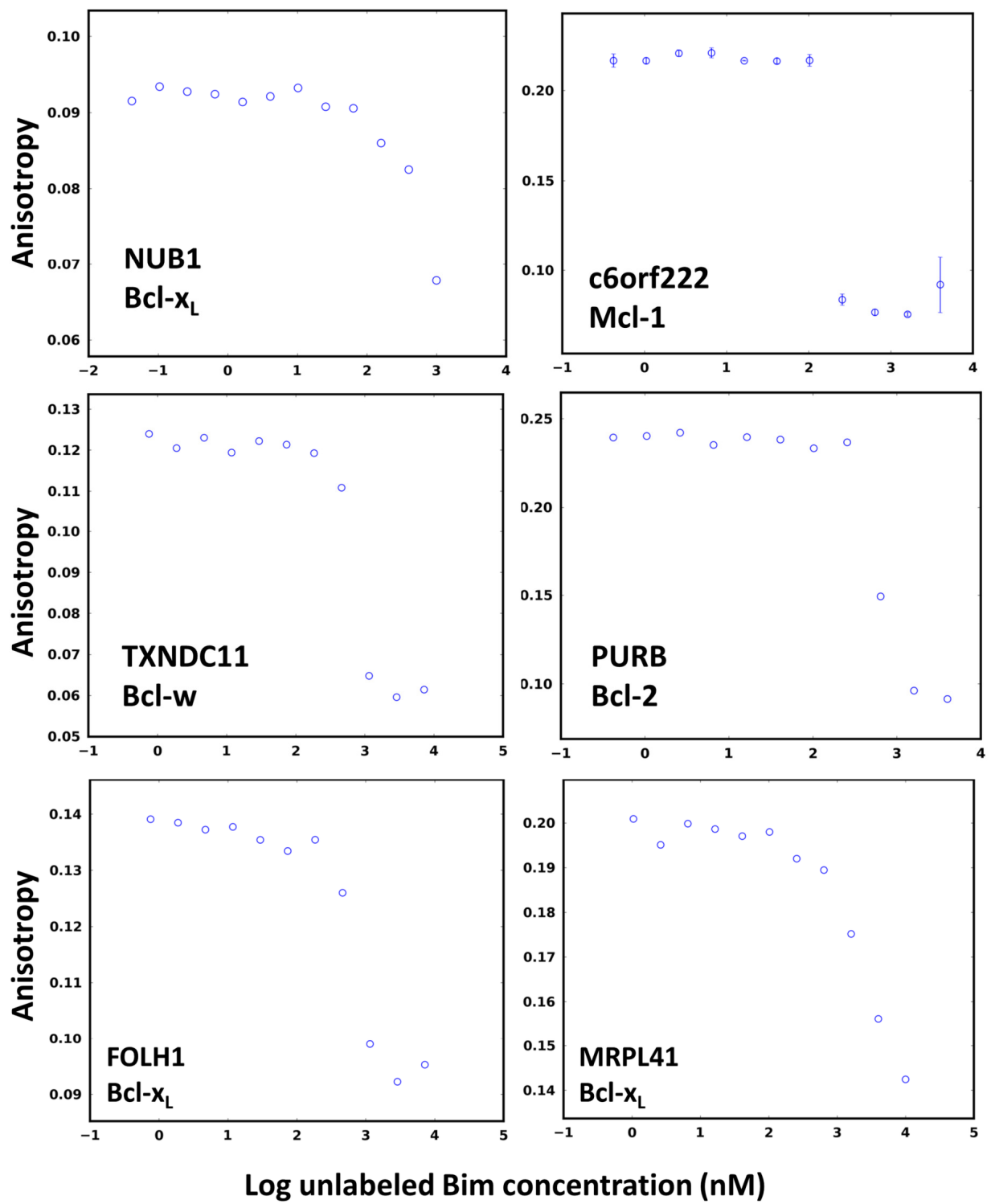

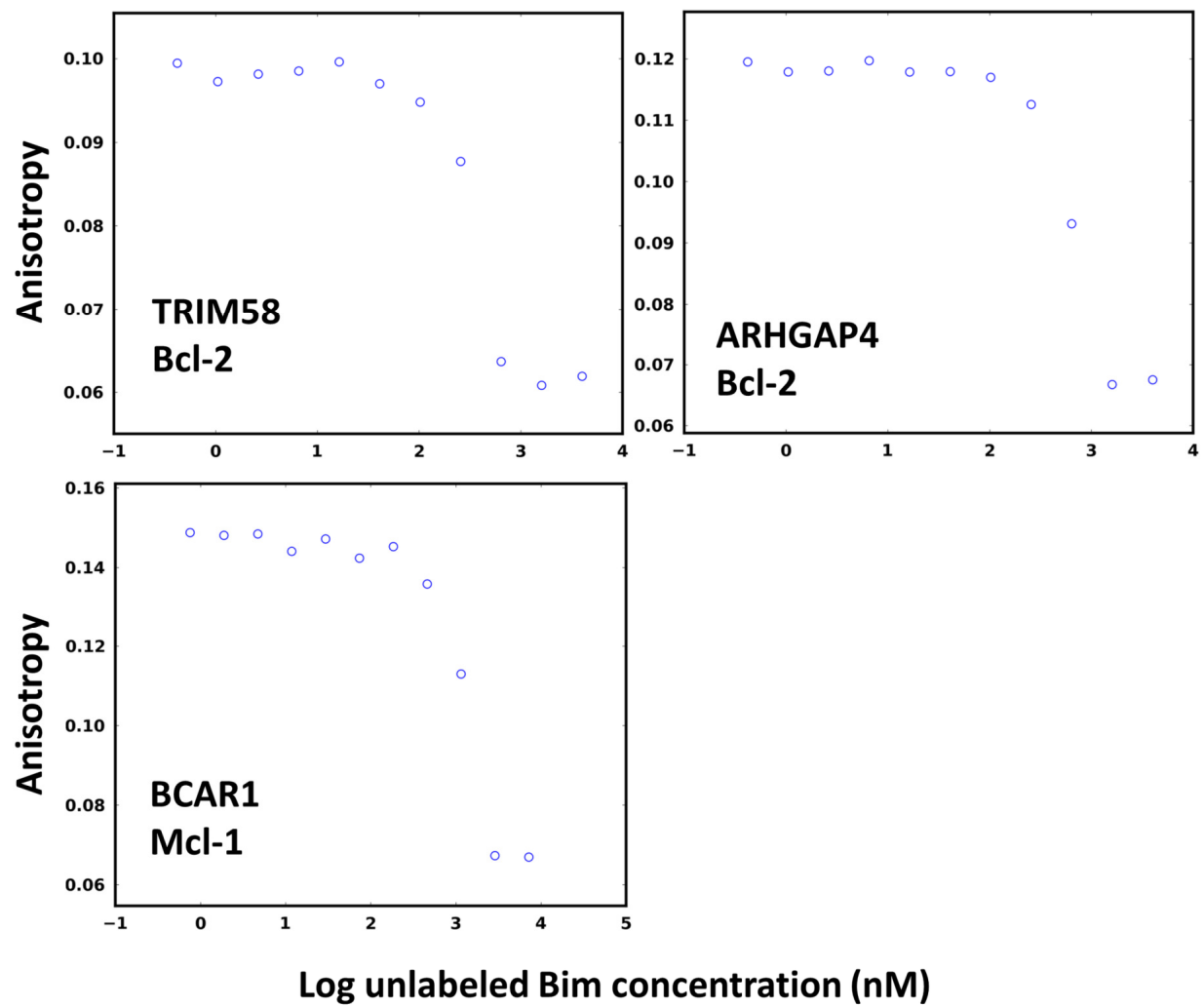

Supplement: Figure S1 — Direct binding of peptides corresponding to predicted BH3 motifs to five human Bcl-2 receptors in solution. Bcl-xL (red), Mcl-1 (blue), Bcl-w (green), Bfl-1 (purple) or Bcl-2 (magenta) were titrated into fluorescein-labeled peptides at a constant concentration of 10 nM. Points are the mean of replicates, and error bars are ±1 standard deviation from the mean of replicates, for illustrative purposes. Curves without error bars are representative curves for cases in which replicates were measured using different concentrations of receptor protein. The KD values and confidence intervals reported in Table S3 resulted from fitting all replicate measurements together. (PDF) [file pcbi.1003693.s001.pdf]
