# Supplementary material for: Genome-Wide Prediction and Validation of Peptides That Bind Human Prosurvival Bcl-2 Proteins
Source: PLoS Comput Biol. 2014 Jun 26;10(6):e1003693. doi: 10.1371/journal.pcbi.1003693 (PMC4072508; doi:10.1371/journal.pcbi.1003693)
Supplement: Figure S2 — Competitive binding of predicted peptides with unlabeled Bim in solution. Fluorescein-labeled peptides were present at a concentration of 10 nM and the receptor concentration varied depending on the strength of binding. Unlabeled Bim BH3 was titrated. The concentration of receptor was 100 nM for PXT1, 25 nM for MCF2L and NBEAL2, 250 nM for SLC19A1, SNTG2 and POFUT2, 2000 nM for PCNA, 250 nM for FOXJ2 and DDX4, 1000 nM for TERT and CASP3, 3000 nM for MCF2L2, 875 nM for TRPM7 and MINA, 3000 for PLEKHH1 and SPNS1, 930 nM for VCAM1, 875 nM for RTEL1, 875 nM for NUB1, 300 nM for c6orf222, 1 µM for TXNDC11, 1 µM for PURB, 1 µM for FOLH1, 1 µM for TRIM58, 1 µM for ARHGAP and 548 nM for BCAR1. MRPL41 was unlabeled and was used to compete with binding of 10 nM labeled Bim to 50 nM Bcl-xL. (PDF) [file pcbi.1003693.s002.pdf]

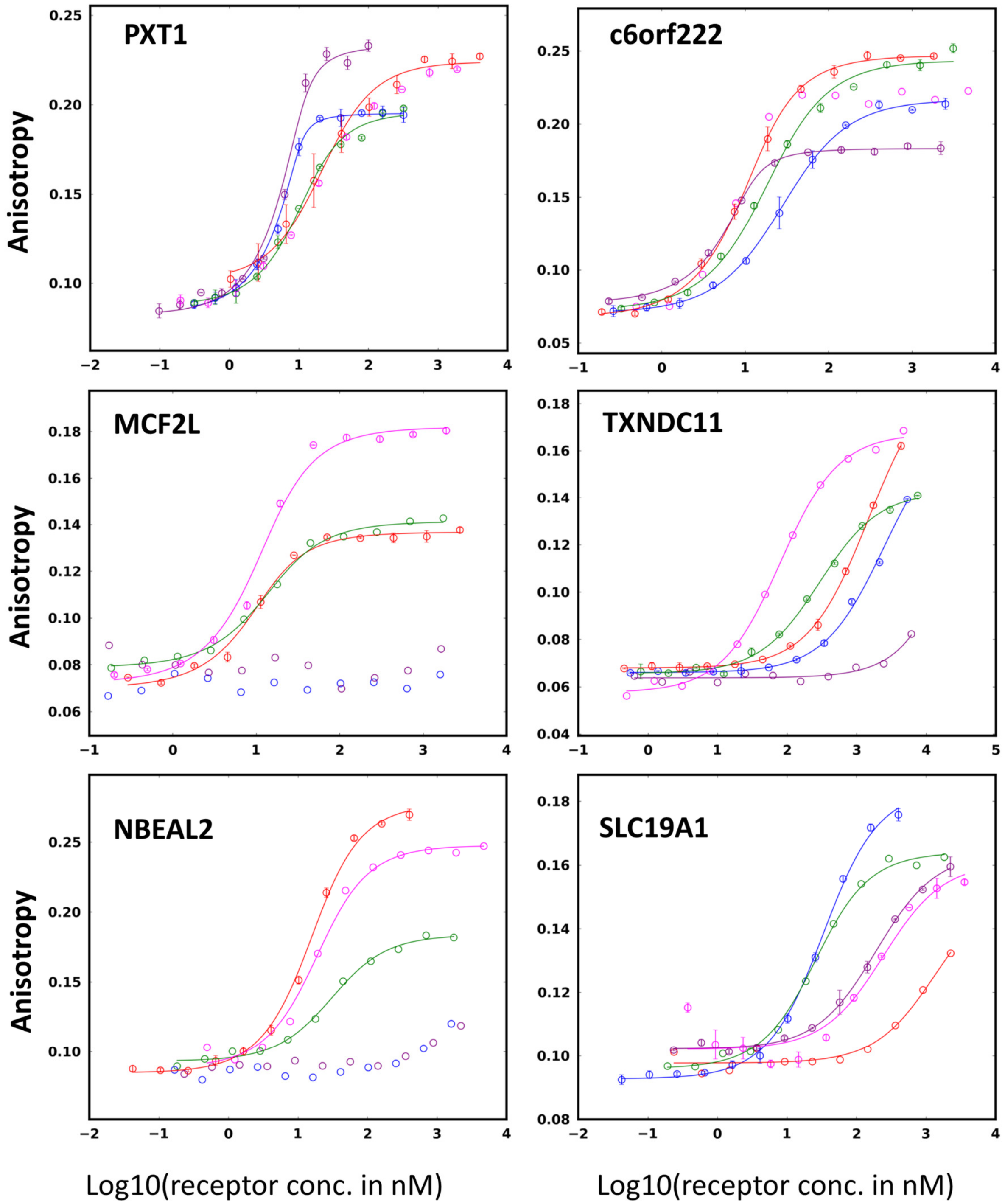
PXT1
c6orf222
MCF2L
TXNDC11
NBEAL2
SLC19A1
Anisotropy
Log10(receptor conc. in nM)

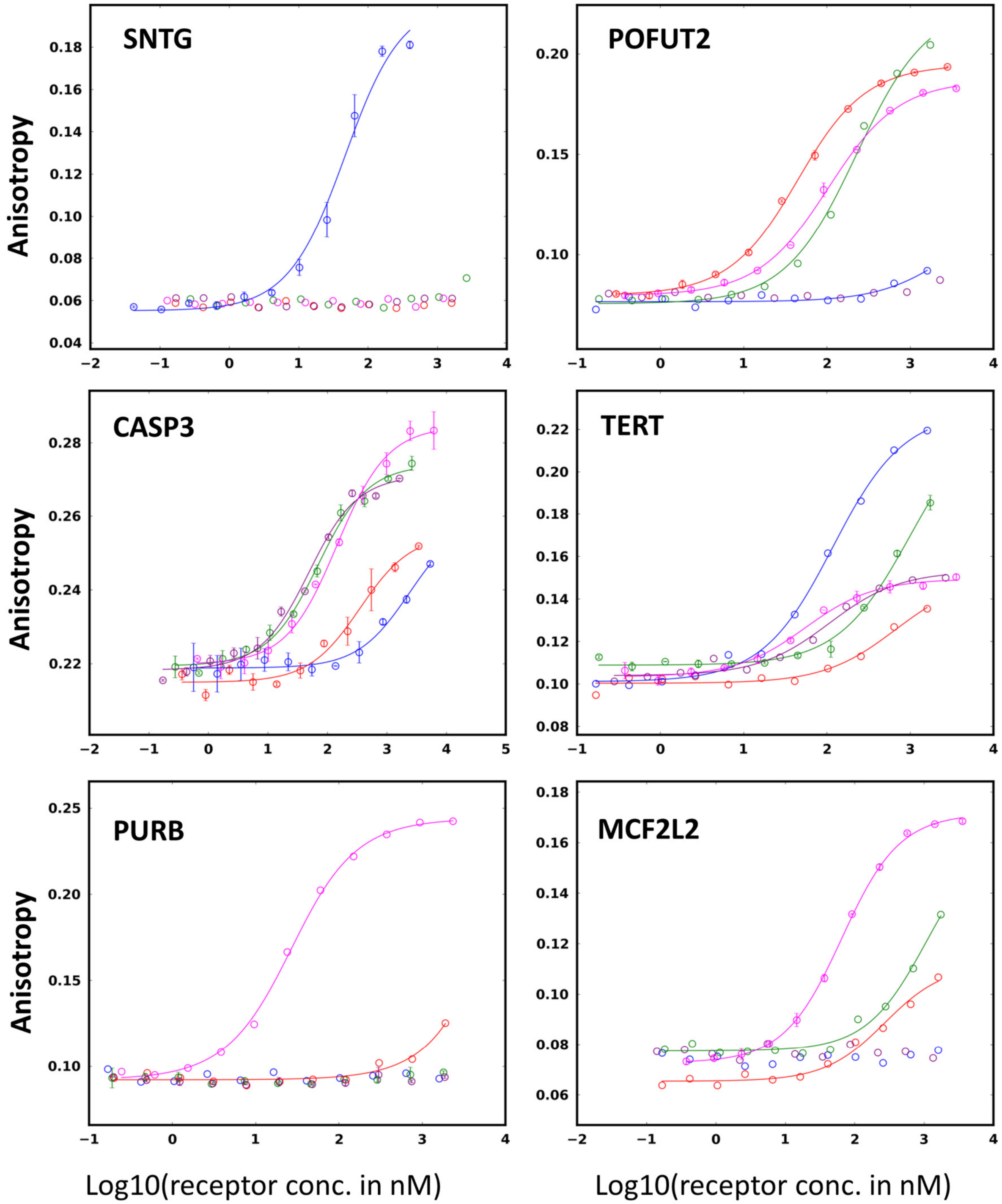
SNTG
POFUT2
CASP3
TERT
PURB
MCF2L2
Anisotropy
Anisotropy
Anisotropy
Log10(receptor conc. in nM)
Log10(receptor conc. in nM)

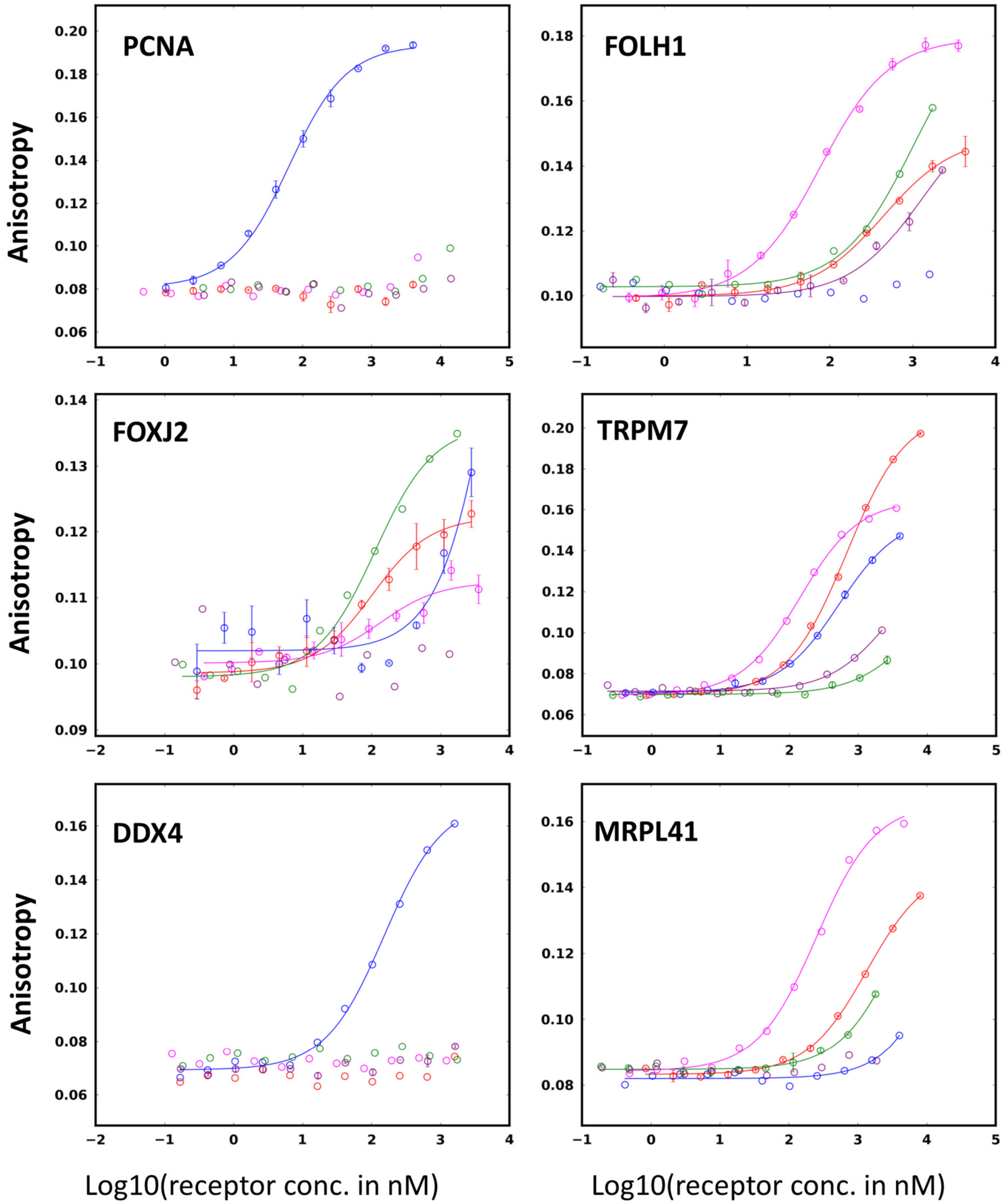
PCNA
FOLH1
FOXJ2
TRPM7
DDX4
MRPL41
Anisotropy
Anisotropy
Anisotropy
Log10(receptor conc. in nM)
Log10(receptor conc. in nM)

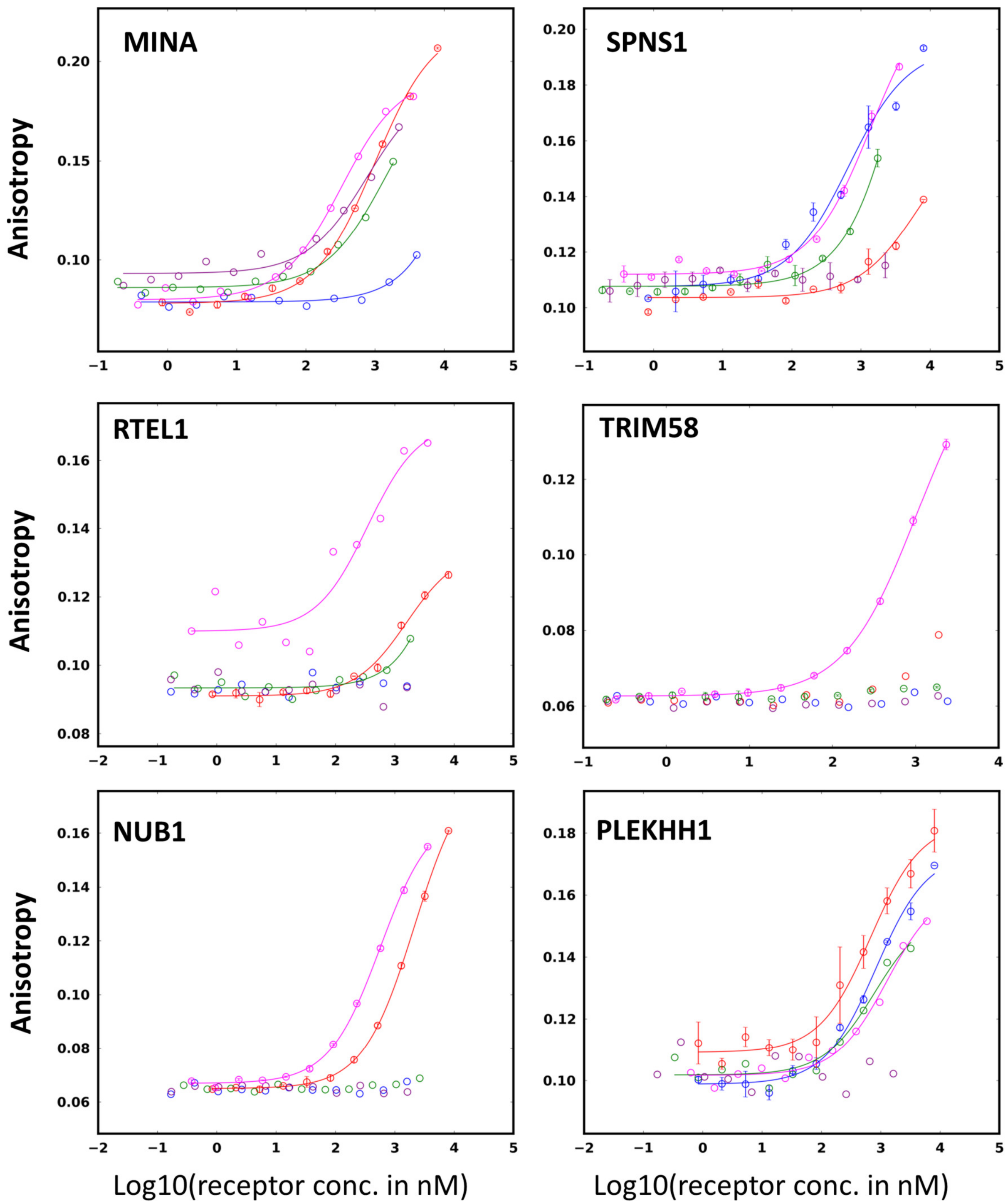

MINA
SPNS1
RTEL1
TRIM58
NUB1
PLEKHH1
Anisotropy
Anisotropy
Anisotropy
Log10(receptor conc. in nM)
Log10(receptor conc. in nM)

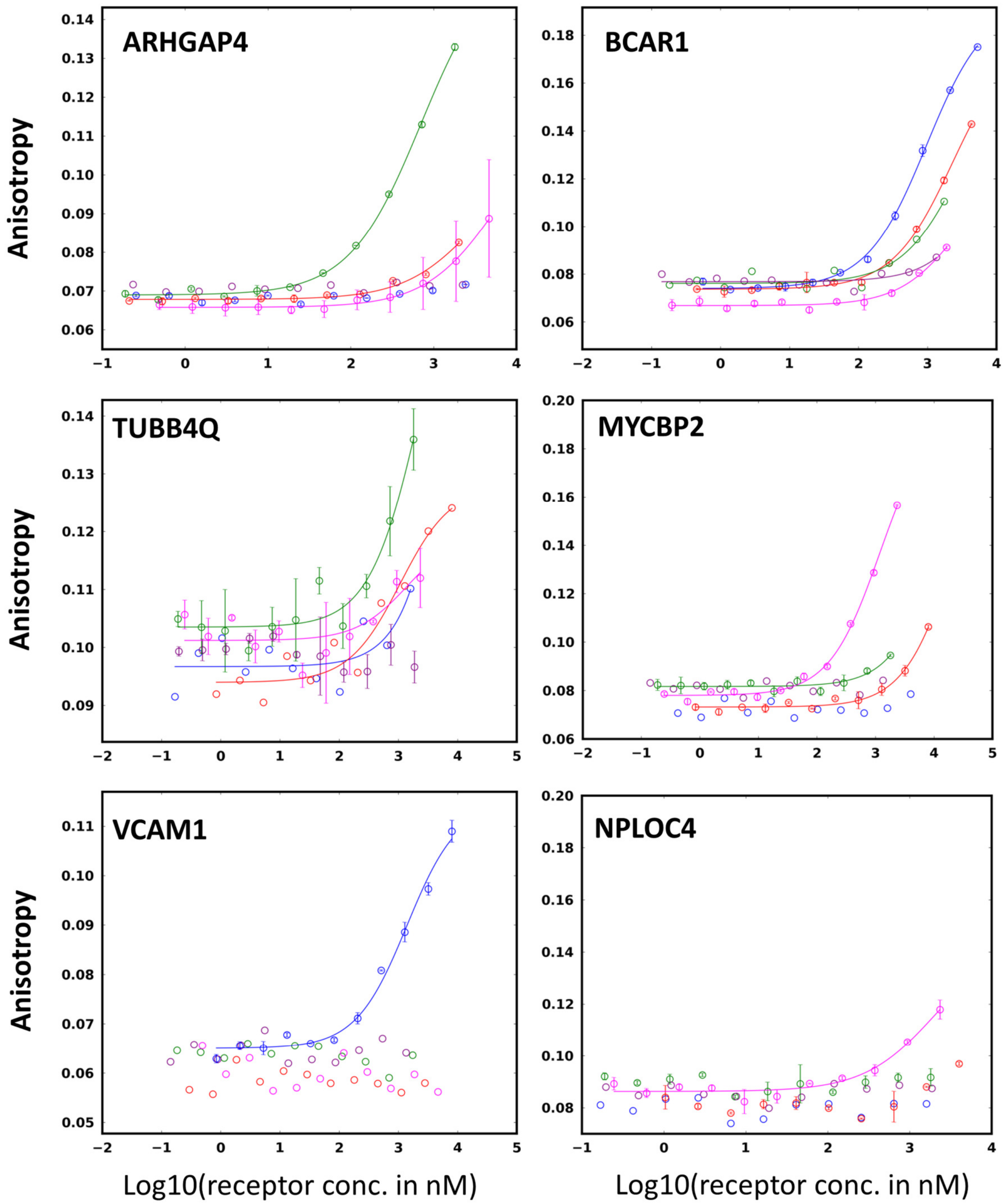
ARHGAP4
BCAR1
TUBB4Q
MYCBP2
VCAM1
NPLOC4
Anisotropy
Anisotropy
Anisotropy
Log10(receptor conc. in nM)
Log10(receptor conc. in nM)

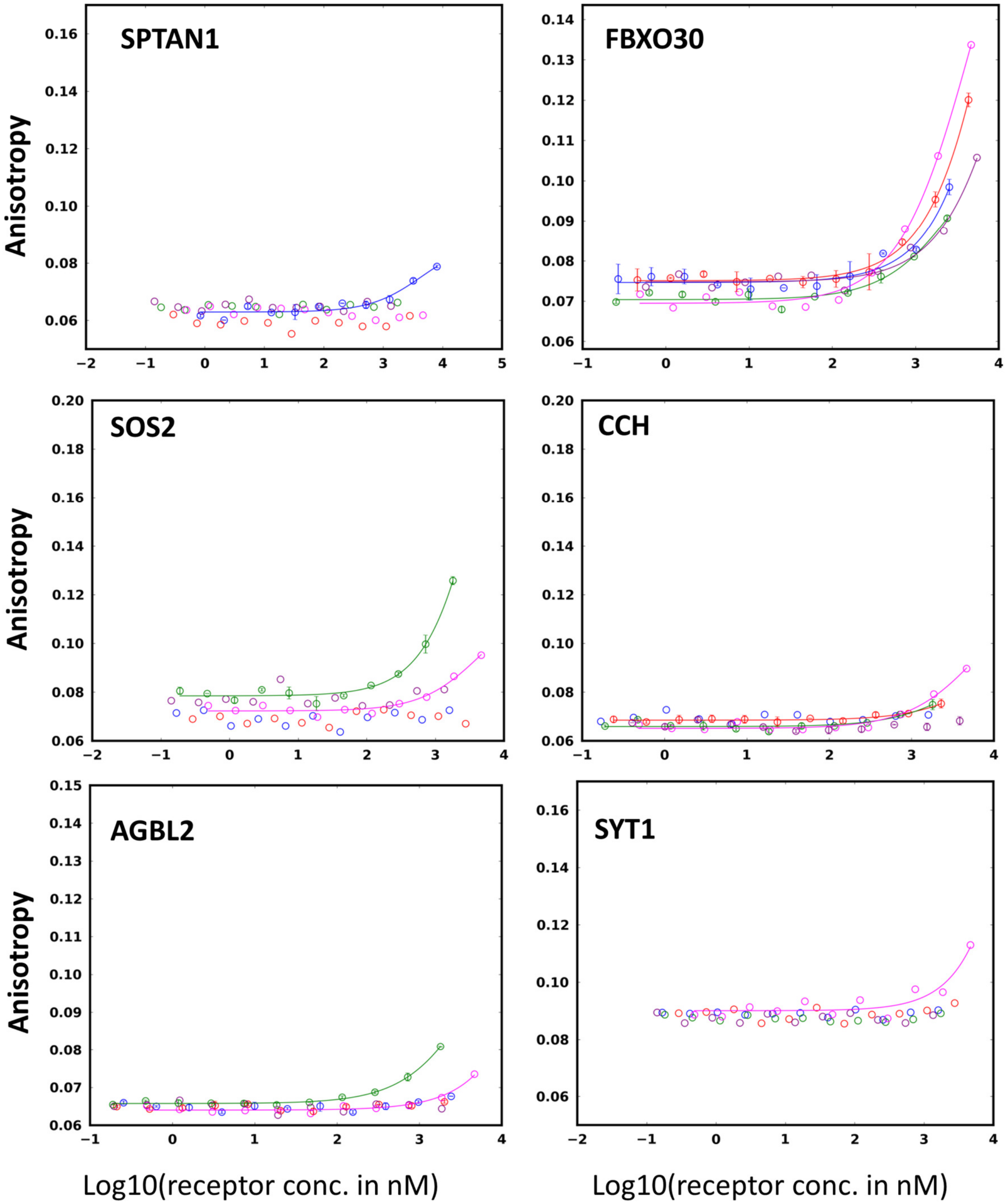
SPTAN1
FBXO30
SOS2
CCH
AGBL2
SYT1
Anisotropy
Anisotropy
Anisotropy
Log10(receptor conc. in nM)
Log10(receptor conc. in nM)
