# Supplementary material for: Genome-Wide Prediction and Validation of Peptides That Bind Human Prosurvival Bcl-2 Proteins
Source: PLoS Comput Biol. 2014 Jun 26;10(6):e1003693. doi: 10.1371/journal.pcbi.1003693 (PMC4072508; doi:10.1371/journal.pcbi.1003693)
Supplement: Table S1 — Conservation of sidechain structure in Bcl-2 complexes. (DOCX) [file pcbi.1003693.s003.docx]

**Table S1. Conservation of sidechain structure in Bcl-2 complexes**

| Complex 1 | | | Complex 2 | | | Comparison | | |
| --- | --- | --- | --- | --- | --- | --- | --- | --- |
| BH3 | Receptor | PDB ID | BH3 | Receptor | PDB ID | Mean RMSD* | Median RMSD* | BH3 % Seq. ID |
| Bim (L3aF) | Bcl-x_L_ | 3io8 | Bad | Bcl-x_L_ | 2bzw | 0.68 | 0.55 | 26 |
| Bim (L3aF) | Bcl-x_L_ | 3io8 | Bim | Bcl-x_L_ | 3fdl | 0.44 | 0.39 | 96 |
| Bad | Bcl-x_L_ | 2bzw | Bim | Bcl-x_L_ | 3fdl | 0.69 | 0.55 | 30 |
| Bim | Bfl-1 | 2vm6 | Noxa | Bfl-1 | 3mqp | 0.3 | 0.23 | 35 |
| Bim | Bfl-1 | 2vm6 | Bak | Bfl-1 | 3i1h | 0.39 | 0.27 | 26 |
| Noxa | Bfl-1 | 3mqp | Bak | Bfl-1 | 3i1h | 0.44 | 0.26 | 26 |
| Bim | Mcl-1 | 2pqk | Bim | Mcl-1 | 2nl9 | 0.18 | 0.14 | 100 |
| Bim | Mcl-1 | 2pqk | Bax | Mcl-1 | 3pk1 | 0.49 | 0.28 | 26 |
| Bim | Mcl-1 | 2nl9 | Bax | Mcl-1 | 3pk1 | 0.55 | 0.3 | 26 |

*Root mean squared difference in atom-atom distances between a receptor-BH3 sidechain pair in complex 1 and the equivalent pair in complex 2. Mean and median values are reported for 40 conserved interacting pairs shared between all receptors.
