# Supplementary material for: Genome-Wide Prediction and Validation of Peptides That Bind Human Prosurvival Bcl-2 Proteins
Source: PLoS Comput Biol. 2014 Jun 26;10(6):e1003693. doi: 10.1371/journal.pcbi.1003693 (PMC4072508; doi:10.1371/journal.pcbi.1003693)
Supplement: Table S2 — SPOT array experiments. (DOCX) [file pcbi.1003693.s004.docx]

**Table S2. SPOT array experiments**

| **Arrays I-IV** | **Array I** | **Array II** | | **Array III** | ***Before IV*** | **Array IV** | **TOTAL** |
| --- | --- | --- | --- | --- | --- | --- | --- |
| Unique peptides | 127 | 176 | 175 | 82 | *560* | 107 | 656 |
| Receptors used for prediction | XMFWB^1^ | X | M | XM | *NA* | XMF | NA |
| Receptors tested | XMFWB | X | M | XM | *NA* | XMF | NA |
| Interactions tested^2^ | 635 | 176 | 175 | 164 | *1150* | 321 | 1471 |
| Control-tested interactions | 60 | 15 | 5 | 164 | *244* | 321 | 565 |
| Successful-control interactions^3^ | 32 | 1 | 1 | 20 | *54* | 57 | 112 |
| Control-tested peptides^4^ | 50 | 15 | 5 | 82 | *152* | 107 | 259 |
| Successful-control peptides^4^ | 24 | 1 | 1 | 17 | *43* | 41 | 84 |
| Enrichment in candidate binders^5^ | 0.48 | 0.07 | 0.2 | 0.21 | *0.28* | 0.38 | 0.32 |
| Interactions w. signal > 5% | 320 | 66 | 69 | 49 | *504* | 278 | 782 |
| Interactions w. signal > 10% | 247 | 47 | 46 | 33 | *373* | 185 | 558 |
| Interactions w. signal > 25% | 162 | 27 | 14 | 17 | *220* | 142 | 362 |
| Peptides w. signal > 5% | 85 | 66 | 69 | 25 | *245* | 105 | 350 |
| Peptides w. signal > 10% | 70 | 47 | 46 | 17 | *180* | 62 | 242 |
| Peptides w. signal > 25% | 51 | 27 | 14 | 8 | *100* | 47 | 147 |
| Solution-tested peptides | 16 | 0 | 0 | 1 | *17* | 19 | 36 |
|  |  |  |  |  |  |  |  |
| **Interactions PSSM_SPOT_ Z-score > 2.0** |  |  |  |  |  |  |  |
| Interactions tested | 319 | 51 | 39 | 148 | *557* | 237 | 794 |
| Interactions w. signal > 5% | 183 | 23 | 20 | 41 | *267* | 193 | 460 |
| Control-tested interactions | 46 | 4 | 4 | 138 | *192* | 214 | 406 |
| Successful-control interactions | 30 | 0 | 1 | 15 | *46* | 46 | 92 |
| Enrichment in candidate binders | 0.65 | 0 | 0.25 | 0.11 | *0.24* | 0.21 | 0.23 |
|  |  |  |  |  |  |  |  |
| **Interactions STATIUM_SC_ Z-score > 2.0** |  |  |  |  |  |  |  |
| Interactions tested | 130 | 19 | 50 | 36 | *235* | 150 | 385 |
| Interactions w. signal > 5% | 82 | 6 | 22 | 18 | *128* | 125 | 253 |
| Control-tested interactions | 18 | 1 | 3 | 34 | *56* | 136 | 192 |
| Successful-control interactions | 14 | 0 | 0 | 4 | *18* | 31 | 49 |
| Enrichment in candidate binders | 0.78 | 0 | 0 | 0.12 | *0.32* | 0.23 | 0.26 |
|  |  |  |  |  |  |  |  |
| **Interactions PSSM_SPOT_+STATIUM_SC_ Z-score^6^ > 2.0** |  |  |  |  |  |  |  |
| Interactions tested | 82 | 13 | 9 | 25 | *129* | 110 | 239 |
| Interactions w. signal > 5% | 57 | 6 | 6 | 12 | *81* | 89 | 170 |
| Control-tested interactions | 15 | 1 | 2 | 23 | *41* | 97 | 138 |
| Successful-control interactions | 14 | 0 | 0 | 2 | *16* | 28 | 44 |
| Enrichment in candidate binders | 0.93 | 0 | 0 | 0.09 | *0.39* | 0.29 | 0.32 |

^1^X = Bcl-x_L_, M = Mcl-1, F = Bfl-1, W = Bcl-w, B = Bcl-2

^2^Unique pairings of peptide and receptor tested on the array.

^3^ These are “candidate array interactions.” Both negative control mutations signals reduced by at least 30% relative to wild-type signal.

^4^Unique peptides with controls tested for *any* receptor.

^5^Successful-control interactions divided by control-tested interactions.

^6^Average of the Z-scores of PSSM_SPOT_ and STATIUM_SC_.
