# Supplementary material for: Genome-Wide Prediction and Validation of Peptides That Bind Human Prosurvival Bcl-2 Proteins
Source: PLoS Comput Biol. 2014 Jun 26;10(6):e1003693. doi: 10.1371/journal.pcbi.1003693 (PMC4072508; doi:10.1371/journal.pcbi.1003693)
Supplement: Table S3 — Best fit KD values and 95% confidence intervals. (DOCX) [file pcbi.1003693.s005.docx]

**Table S3. Best-fit K_D_ values and 95% confidence intervals^1^**

|  | **Bcl-x_L_** | | | **Bcl-w** | | | **Bcl-2** | | | **Mcl-1** | | | **Bfl-1** | | |
| --- | --- | --- | --- | --- | --- | --- | --- | --- | --- | --- | --- | --- | --- | --- | --- |
| **PXT1** | **8** | *6* | *11* | **5** | *4* | *6* | **14** | *12* | *16* | **0.9** | *0.5* | *1.5* | **1** | *0.6* | *1.5* |
| **C6orf222** | **5** | *5* | *6* | **13** | *11* | *15* | **0.3** | *0.1* | *1.3* | **23** | *19* | *27* | **1** | *1* | *1.5* |
| **MCF2L** | **4** | *2* | *6* | **7** | *5* | *9* | **6** | *5* | *8* | X4000 | ***NA*** | ***NA*** | X4100 | ***NA*** | ***NA*** |
| **NBEAL2** | **10** | *9* | *11* | **29** | *24* | *35* | **16** | *13* | *19* | 3806 | *1979* | *7323* | 3273 | *1134* | *9453* |
| **SLC19A1** | 1458 | *694* | *3065* | **18** | *15* | *21* | **237** | *156* | *361* | **22** | *19* | *27* | **182** | *159* | *211* |
| **SPNS1^2^** | **96** | *36* | *261* | **871** | *86* | *8820* | **28** | *15* | *54* | **19** | *7* | *50* | **278** | *101* | *760* |
| **SNTG2** | X4000 | ***NA*** | ***NA*** | X6562 | ***NA*** | ***NA*** | X3045 | ***NA*** | ***NA*** | **22** | *20* | *41* | X4068 | ***NA*** | ***NA*** |
| **POFUT2** | **39** | *37* | *43* | **98** | *60* | *163* | **98** | *90* | *107* | >10^4^ | *6707* | *>10^5^* | X 2280 | ***NA*** | ***NA*** |
| **PURB** | >10^5^ | *>10^4^* | *>10^6^* | X1800 | ***NA*** | ***NA*** | **34** | *27* | *45* | X4000 | ***NA*** | ***NA*** | X4665 | ***NA*** | ***NA*** |
| **CASP3** | **295** | *211* | *413* | **64** | *53* | *79* | **264** | *145* | *481* | 1190 | *675* | *2099* | **43** | *35* | *54* |
| **TERT** | 1138 | *681* | *1904* | 992 | *773* | *1274* | **49** | *38* | *64* | **69** | *52* | *94* | **99** | *81* | *122* |
| **PCNA** | X10^4^ | ***NA*** | ***NA*** | >10^4^ | ***NA*** | ***NA*** | >10^5^ | ***NA*** | ***NA*** | **59** | *51* | *69* | X10^4^ | ***NA*** | ***NA*** |
| **MCF2L2** | **292** | *153* | *556* | 1116 | *819* | *1523* | **60** | *56* | *65* | X4000 | ***NA*** | ***NA*** | X3367 | ***NA*** | ***NA*** |
| **FOLH1** | **483** | *391* | *599* | 907 | *712* | *1156* | **70** | *61* | *81* | X 4000 | ***NA*** | ***NA*** | 1600 | *1172* | *2185* |
| **FOXJ2** | **103** | *79* | *134* | **85** | *65* | *113* | **130** | *70* | *242* | >10^4^ | *2776* | *>10^4^* | X3367 | ***NA*** | ***NA*** |
| **TXNDC11** | 1486 | *1346* | *1639* | **249** | *220* | *282* | **86** | *76* | *97* | 2379 | *2112* | *2681* | >10^4^ | *7642* | *>10^5^* |
| **TRPM7** | 679 | *567* | *814* | 2489 | *397* | *>10^4^* | **124** | *115* | *135* | 592 | *432* | *812* | 1904 | *443* | *8188* |
| **DDX4** | X4000 | ***NA*** | ***NA*** | X4350 | ***NA*** | ***NA*** | X3045 | ***NA*** | ***NA*** | **142** | *119* | *170* | X3367 | ***NA*** | ***NA*** |
| **MRPL41** | 1239 | *1107* | *1389* | 4324 | *2109* | *8867* | **191** | *156* | *236* | >10^5^ | *>10^4^* | *>10^6^* | X4665 | ***NA*** | ***NA*** |
| **MINA** | 1006 | *933* | *1086* | 1232 | *951* | *1596* | **266** | *231* | *307* | >10^4^ | *5494* | *>10^5^* | 1134 | *542* | *2005* |
| **RTEL1** | 1377 | *1112* | *1705* | >10^5^ | *4503* | *>10^6^* | **342** | *169* | *697* | X4000 | ***NA*** | ***NA*** | X4000 | ***NA*** | ***NA*** |
| **TRIM58^3^** | 4552 | *467* | *>10^4^* | **361** | *133* | *984* | 975 | *805* | *1182* | X6071 | ***NA*** | ***NA*** | X4665 | ***NA*** | ***NA*** |
| **NUB1** | 2715 | *1938* | *3807* | X4000 | ***NA*** | ***NA*** | 562 | *528* | *600* | X10^4^ | ***NA*** | ***NA*** | X4000 | ***NA*** | ***NA*** |
| **PLEKHH1** | 650 | *441* | *960* | 1398 | *726* | *2694* | 1500 | *1101* | *2045* | 977 | *741* | *1290* | X5680 | ***NA*** | ***NA*** |
| **ARHGAP4^4^** | 3063 | *428* | *>10^4^* | 749 | *611* | *919* | 6091 | *1457* | *>10^4^* | X2429 | ***NA*** | ***NA*** | X5595 | ***NA*** | ***NA*** |
| **BCAR1** | 2538 | *2157* | *2987* | 3608 | *2088* | *6235* | 2987 | *1459* | *6117* | 912 | *818* | *1018* | 4685 | *835* | *>10^4^* |
| **VCAM1** | X7000 | ***NA*** | ***NA*** | X4350 | ***NA*** | ***NA*** | X3045 | ***NA*** | ***NA*** | 1139 | *887* | *1463* | X3368 | ***NA*** | ***NA*** |
| **MYCBP2** | > 10^4^ | *>10^4^* | *>10^4^* | 4065 | *920* | *>10^4^* | 1181 | *1015* | *1375* | X10^4^ | ***NA*** | ***NA*** | X3370 | ***NA*** | ***NA*** |
| **TUBB4Q** | 2054 | *826* | *5108* | 2492 | *844* | *7361* | 1409 | *319* | *6231* | 1463 | *393* | *5449* | X4665 | ***NA*** | ***NA*** |
| **CCH** | X5723 | ***NA*** | ***NA*** | 1915 | *698* | *5258* | 7133 | *3459* | *>10^4^* | X4000 | ***NA*** | ***NA*** | X9615 | ***NA*** | ***NA*** |
| **NPLOC4** | >10^5^ | *4580* | *>10^6^* | X4500 | ***NA*** | ***NA*** | 2058 | *1085* | *3905* | X4000 | ***NA*** | ***NA*** | X4665 | ***NA*** | ***NA*** |
| **FBXO30** | >10^4^ | *9140* | *>10^4^* | 3282 | *1792* | *6011* | 2564 | *1266* | *5193* | >10^4^ | *4350* | *>10^5^* | >10^4^ | *>10^4^* | *>10^5^* |
| **SOS2** | X6900 | ***NA*** | ***NA*** | 5379 | *1676* | *>10^4^* | 3286 | *1264* | *8545* | X4000 | ***NA*** | ***NA*** | X3294 | ***NA*** | ***NA*** |
| **SPTAN1** | X7000 | ***NA*** | ***NA*** | X4350 | ***NA*** | ***NA*** | X3045 | ***NA*** | ***NA*** | 3849 | *2225* | *6659* | X3370 | ***NA*** | ***NA*** |
| **AGBL2** | X4000 | ***NA*** | ***NA*** | 5868 | *3081* | *>10^4^* | >10^5^ | *>10^4^* | *>10^6^* | X5025 | ***NA*** | ***NA*** | X4665 | ***NA*** | ***NA*** |
| **SYT1** | X6900 | ***NA*** | ***NA*** | X4350 | ***NA*** | ***NA*** | >10^5^ | *4679* | *>10^6^* | X4000 | ***NA*** | ***NA*** | X3367 | ***NA*** | ***NA*** |

**^1^**Best-fit K_D_ values determined by combining all replicate titration curves and fitting to a direct binding model (see Methods). The standard devation of the K_D_ fit parameter was converted to a student t-value to account for the small number of data points and the 95% confidence interval was taken from a t-distribution. The first value listed is the K_D_ and the next two values are the upper and lower bounds of the 95% confidence interval. Values in blue mark best-fit K_D_ values for which the upper-limit of the 95% confidence interval is less than 500 nM. If the best fit K_D_ is greater than 10^4^ nM, the K_D_ is colored red. Values with an X followed by a number indicate the highest concentration (the number, in nM) for which signal was measured yet no binding was detected.

^2^SPNS1 values are from fitting the raw fluorescence data (see Methods)

^3^Upper baseline signal is low and close to the lower baseline for Bcl-w binding.

^4^Fitting the raw fluorescence signal suggested lower K_D_ values for Bfl-1 binding, but the data was very noisy.
