# Supplementary material for: Genome-Wide Prediction and Validation of Peptides That Bind Human Prosurvival Bcl-2 Proteins
Source: PLoS Comput Biol. 2014 Jun 26;10(6):e1003693. doi: 10.1371/journal.pcbi.1003693 (PMC4072508; doi:10.1371/journal.pcbi.1003693)
Supplement: Table S4 — Sequences of weak peptide binders. (DOCX) [file pcbi.1003693.s006.docx]

**Table S4. Sequences of weak peptide binders.**

|  |  | **position of peptide**  **residue 1 in protein** |
| --- | --- | --- |
| TDENKKVGLALKDLAKQYSDRLECCE | NUB1 | 28 |
| RYRHGAPAEQLRHLADMLTTKWATLQ | PLEKHH1 | 1179 |
| EVLAGPLAQRLSHIAEDVGRLVKKSR | ARHGAP4 | 122 |
| SKFVILSAHKLVFIGDTLSRQAKAAD | BCAR1 | 782 |
| FTGNNTAVQELKRVSEQFTATFRRKA | TUBB4Q | 367 |
| AIILCNVCGNLCTDCDRFLHLHRRTK | MYCBP2 | 4233 |
| KIETTPESRYLAQIGDSVSLTCSTTG | VCAM1 | 26 |
| ATKRETAATFLKKVAKEFGFQNNGFS | NPLOC4 | 20 |
| QRKHEGFERDLAALGDKVNSLGETAE | SPTAN1 | 1272 |
| LCQLSCVSKLMRDVCGSLLQSRGMVI | FBXO30 | 632 |
| LMARPAVALHFQSIADGFKEAVRYVL | SOS2 | 312 |
| FVGYNSTGAELRHWSDMLANPRRPIA | SYT1 | 378 |
