# Supplementary material for: Genome-Wide Prediction and Validation of Peptides That Bind Human Prosurvival Bcl-2 Proteins
Source: PLoS Comput Biol. 2014 Jun 26;10(6):e1003693. doi: 10.1371/journal.pcbi.1003693 (PMC4072508; doi:10.1371/journal.pcbi.1003693)
Supplement: Table S5 — Summary of structures of known and predicted BH3 motifs. (DOCX) [file pcbi.1003693.s007.docx]

**Table S5. Summary of structures of known and predicted BH3 motifs.**

| Puma | Intrinsically disordered* |  |  |
| --- | --- | --- | --- |
| Bim | Intrinsically disordered |  |  |
| Noxa | Intrinsically disordered * |  |  |
| Bad | Intrinsically disordered |  |  |
| Bak | α, 2yv6 |  |  |
| Bax | α, 1f16 |  |  |
| Hrk | Intrinsically disordered |  |  |
| Bmf | Intrinsically disordered |  |  |
| Bik | α* |  |  |
| Bid | α, 2bid |  |  |
| Mule | Intrinsically disordered * |  |  |
| Beclin | α* |  |  |
| Bok | α* | **Apop. refs.** |  |
| PXT1 | α/β* | [1] |  |
| c6orf222 | Intrinsically disordered * |  |  |
| MCF2L | α, 1cun^ǂ^ |  |  |
| TXNDC11 | α* |  |  |
| NBEAL2 | α* |  |  |
| SLC19A1 | α/β* |  |  |
| SNTG2 | α/β, 1z87^ǂ^ |  |  |
| POFUT2 | α/β, 4ap5 |  |  |
| CASP3 | α/β, 1cp33 | [2] |  |
| TERT | α* | [3] |  |
| PURB | α/β, 3k44 |  |  |
| MCF2L2 | α, 1cun^ǂ^ |  |  |
| PCNA | α/β, 1axc | [4] |  |
| FOLH1 | α/β, 3bi1 |  |  |
| FOXJ2 | α/β, 1e17^ǂ^ |  |  |
| TRPM7 | Coiled-coil tetramer, 3e7k | [5] |  |
| DDX4 | α/β, 2db3 |  |  |
| MRPL41 | N-term. tag on α/β | [6] |  |
| MINA | α/β, 2xdv |  |  |
| SPNS1 | α/β transmembrane, 1pw4^ǂ^ | [7] |  |
| RTEL1 | α/β, 3crv^ǂ^ |  |  |
| TRIM58 | α/β* |  |  |
| BCAR1 | α, 3t6g* | [8] |  |

*****Prediction. Secondary structure predicted using Psipred. If Psipred predicts coil, intrinsic disorder (ID) is predicted using DisEmbl and Disprot (see Methods).

^ǂ^PDB ID based on homolog from CDD (see Methods).
