# Supplementary material for: Genome-Wide Prediction and Validation of Peptides That Bind Human Prosurvival Bcl-2 Proteins
Source: PLoS Comput Biol. 2014 Jun 26;10(6):e1003693. doi: 10.1371/journal.pcbi.1003693 (PMC4072508; doi:10.1371/journal.pcbi.1003693)
Supplement: Table S6 — Sequences of prosurvival receptor constructs. (DOCX) [file pcbi.1003693.s008.docx]

**Table S6. Sequences of prosurvival receptor constructs**

| myc-Mcl-1 SPOT array & FP | GSEQKLISEEDLGGSGGTSDELYRQSLEIISRYLREQATGAKDTKPMGRSGATSRKALETLRRVGDGVQRNHETAFQGMLRKLDIKNEDDVKSLSRVMIHVFSDGVTNWGRIVTLISFGAFVAKHLKTINQESCIEPLAESITDVLVRTKRDWLVKQRGWDGFVEFFHVEDLEGG |
| --- | --- |
| myc-Bcl-x_L_ SPOT array & FP | GSEQKLISEEDLGGSGGTSMSQSNRELVVDFLSYKLSQKGYSWSQFSDVEENRTEAPEGTESEMETPSAINGNPSWHLADSPAVNGATGHSSSLDAREVIPMAAVKQALREAGDEFELRYRRAFSDLTSQLHITPGTAYQSFEQVVNELFRDGVNWGRIVAFFSFGGALCVESVDKEMQVLVSRIAAWMATYLNDHLEPWIQENGGWDTFVELYGNNAAAESRKGQER |
| myc-Bcl-w SPOT array & FP | GSEQKLISEEDLGGSGGTSMATPASAPDTRALVADFVGYKLRQKGYVCGAGPGEGPAADPLHQAMRAAGDEFETRFRRTFSDLAAQLHVTPGSAQQRFTQVSDELFQGGPNWGRLVAFFVFGAALCAESVNKEMEPLVGQVQEWMVAYLETRLADWIHSSGGWAEFTALYGDGALEEARRLRE |
| myc- Bfl-1 SPOT array & FP | GSEQKLISEEDLGGSGGTSMTDCEFGYIYRLAQDYLQCVLQIPQPGSGPSKTSRVLQNVAFSVQKEVEKNLKSCLDNVNVVSVDTARTLFNQVMEKEFEDGIINWGRIVTIFAFEGILIKKLLRQQIAPDVDTYKEISYFVAEFIMNNTGEWIRQNGGWENGFVKKFEPK |
| myc-Bcl-2 SPOT array | SEQKLISEEDLGGSGGTSMAHAGRTGYDNREIVMKYIHYKLSQRGYEWDAGDVGAAPPGAAPAPGIFSSQPGHTPHPAASRDPVARTSPLQTPAAPGAAAGPALSPVPPVVHLTLRQAGDDFSRRYRRDFAEMSSQLHLTPFTARGRFATVVEELFRDGVNWGRIVAFFEFGGVMCVESVNREMSPLVDNIALWMTEYLNRHLHTWIQDNGGWDAFVELYGPSMRPLFDFSWLSL |
| myc- Bcl-2 FP | SEQKLISEEDLGGSGGTSMAHAGRTGYDNREIVMKYIHYKLSQRGYEWDAGDVGAAPPGAAPAPGIFSSQVVHLTLRQAGDDFSRRYRRDFAEMSSQLHLTPFTARGRFATVVEELFRDGVNWGRIVAFFEFGGVMCVESVNREMSPLVDNIALWMTEYLNRHLHTWIQDNGGWDAFVELYGPS |
| LUMIER Bcl-x_L_ | MGGSGGTSMSQSNRELVVDFLSYKLSQKGYSWSQFSDVEENRTEAPEGTESEMETPSAINGNPSWHLADSPAVNGATGHSSSLDAREVIPMAAVKQALREAGDEFELRYRRAFSDLTSQLHITPGTAYQSFEQVVNELFRDGVNWGRIVAFFSFGGALCVESVDKEMQVLVSRIAAWMATYLNDHLEPWIQENGGWDTFVELYGNNAAAESRKGQER |
| LUMIER Mcl-1 | MGGSGGTSDELYRQSLEIISRYLREQATGAKDTKPMGRSGATSRKALETLRRVGDGVQRNHETAFQGMLRKLDIKNEDDVKSLSRVMIHVFSDGVTNWGRIVTLISFGAFVAKHLKTINQESCIEPLAESITDVLVRTKRDWLVKQRGWDGFVEFFHVEDLEGG |
