# Supplementary material for: Genome-Wide Prediction and Validation of Peptides That Bind Human Prosurvival Bcl-2 Proteins
Source: PLoS Comput Biol. 2014 Jun 26;10(6):e1003693. doi: 10.1371/journal.pcbi.1003693 (PMC4072508; doi:10.1371/journal.pcbi.1003693)
Supplement: Text S1 — Conservation of sidechain structure in Bcl-2 complexes. (DOCX) [file pcbi.1003693.s012.docx]

**SI Results: Conservation of sidechain structure in Bcl-2 complexes**

The derivation of STATIUM_SC_ assumes that the geometry of an interacting sidechain pair in a protein-peptide complex does not change substantially when the peptide sequence changes. To evaluate the extent to which this is true for Bcl-2 family complexes, we compared three different crystal structures for each of Bcl-x_L_, Mcl-1 and Bfl-1 (Table S1). Eight complex-complex comparisons involved the same receptor bound to two different peptides, and one involved two structures of Mcl-1 bound to Bim BH3. BH3 peptide sequence identities are given in Table S1.

STATIUM_SC_ compares the geometry of two side-chain pairs using the root mean squared difference in atom-atom distances (RMSD) between the full complement of sidechain atoms in a receptor residue and the Cα/Cβ atoms of the peptide residue. Pairs are considered to match if the RMSD is below 0.4 Å. We used the same metric to compare the sidechain geometries for 40 receptor-peptide residue pairs found in all 9 pairs of Bcl-2 protein complexes. The mean and median RMSD values are reported in Table S1. The mean and median values can be as low as 0.3 and 0.23 Å, respectively, for the same receptor bound to two peptides with low sequence identity. Average values for all structure comparisons are 0.46 and 0.33 Å. We also noted that the comparison of two Mcl-1/Bim complexes (2PQK and 2NL9) resulted in mean and median RMSD values of 0.18 and 0.14 Å, providing an estimate of a lower bound for the RMSD comparison. The observation that most of the conformational changes that accompany changes in peptide sequence are within the cutoff used to assign matching geometries in STATIUM_SC_ is consistent with the good performance we observe for this model.
